# Techniques for In Situ Monitoring the Performance of Organic Coatings and Their Applicability to the Pre-Finished Steel Industry: A Review

**DOI:** 10.3390/s21196334

**Published:** 2021-09-22

**Authors:** Tim Savill, Eifion Jewell

**Affiliations:** Materials Research Center, College of Engineering, Bay Campus, Swansea University, Crymlyn Burrows, Swansea SA1 8EN, UK; E.Jewell@swansea.ac.uk

**Keywords:** corrosion sensing, organic coating sensing, in situ, pre-finished steel

## Abstract

A review is carried out in this paper into techniques that currently exist for, of have the potential to be used for, monitoring the performance of organic coating. Specific attention is paid to the applicability of each method to pre-finished steel used in the construction industry as these are rarely monitored in situ and their expected performance is often only estimated from lab-based accelerated corrosion testing. Monitoring could allow more accurate estimates of building cladding lifespan and required maintenance schedules; provide customers with active performance data; additionally, with a better understanding of performance, more appropriate coatings or coating weights could be selected for a construction project, offering economic benefits as part of smart building developments. An introduction to coatings, their use for corrosion protection, failure mechanisms, and relevant monitoring techniques is given before current assessment techniques are described in terms of their working principles. Examples of recent work are presented for the techniques that have been investigated for monitoring or directly relatable purposes. The review concludes that there are several good reasons why an optimum corrosion monitoring technology does not currently exist, however, promising research is emerging in the field of wireless and embedded sensor design which is providing optimistic results.

## 1. Introduction

The cost of corrosion is extremely extensive with the combined direct and indirect impact of corrosion being estimated by several studies to be approximately 6–8% of GDP [1,2,3]. The construction industry represents a large share of this financial burden, and it was reported that the cost due to infrastructure corrosion in the UK in 1970 was estimated to be GBP 250 million [3].

It has been suggested by some studies, that the reason for the high cost of corrosion is due, in part, to poor selection of protective measures [4]. Hence, one widely suggested strategy for corrosion protection is to ‘develop advanced life-prediction and performance-assessment methods’ and to move to a greater degree of corrosion monitoring [2].

Organic coatings are widely used in the construction industry for corrosion protection. For example, in 2017 the EU produced 153 million metric tonnes of hot rolled steel, of which 4 million metric tonnes was organically coated [5]. A significant quantity of this organically coated (pre-finished) steel is used for building cladding purposes which provide a convenient versatile building material to provide an aesthetic and weather resistant surface. These cladding panels face harsh conditions in use and there are large aesthetic and structural ramifications if they fail. Despite this, coatings are rarely monitored in situ and expected performance of the overall building envelope is often only estimated from lab-based accelerated corrosion testing. It is important to note that monitoring aims to detect coating degradation or corrosion intensity and propagation rate and is not a preventative solution. However, their application should, in theory, allow far better understanding as to the health of an asset and hence reduce failure rate and minimise costs.

The oil and gas industry have long been aware of the benefits of monitoring of pipelines [6] and routinely use techniques such as ultrasonic testing and intelligent pigging. Currently, however, they are the only significant industry with commonly used, commercially viable, monitoring methods. This is despite the realisation of the importance of asset monitoring by other sectors and emerging research in these areas. This review will aim to evaluate the current state of the art research into specific sensors for pre-finished steel monitoring as well as reviewing other coating monitoring methods that may be considered for this application. This review will encompass the spectrum of sensor technologies from those in use to those which are conceptually viable, but which have to be researched for the pre-finished steel sector.

## 2. Coatings for Corrosion Protection

A coating is defined by ISO 4618 [7] to be a “layer formed from a single or multiple applications of a coating material to a substrate”. This coating material is said to be a “Product, in liquid, paste or powder form, that, when applied to a substrate, forms a layer possessing protective, decorative and/or other specific properties”. The substrate is simply the “surface to which a coating material is applied to”. In the case of pre-finished steel, the substrate is the steel body of the panel, and the coating refers to a complex system of organic and metal layers which are deposited onto this substrate. The use of both metallic and organic coating layers is widespread in the coating industry and aims to provide the best possible protection by utilising the advantage of both systems in conjunction. As organic coatings form the top layers of a coating system they could be regarded as the ’First line of defence’ and hence face the most demanding conditions.

Metallic coatings can offer galvanic protection of a substrate. Perhaps the most common example of this involves applying a zinc coating to a steel substrate, carried out in a process called galvanising. Zinc is more electroactive than iron so corrodes preferentially to the steel, but it also forms an insoluble corrosion product which provides a further layer of protection. This layer is regarded as ‘self-healing’ as if breached through mechanical damage the zinc will corrode producing further insoluble product that can repair the zinc layer. This type of protection is often called sacrificial protection and can be incorporated into organic paint systems through the use of metal powders [8].

Other metals that are not more electroactive than iron may also be used as coatings such as nickel or chromium. These are used commonly for aesthetic reasons and solely provide a barrier to the environment. Materials that are very inert such as tin or that form inert oxide layers such as aluminium are also often used to provide durable coating barrier layers.

Organic coatings, or paints, protect a substrate by removing one or more of the four components required for aqueous corrosion [8] and hence prevent the electrochemical reaction. This can be achieved through a variety of ways. At the most basic level they are barriers between an aggressive environment and a metal to be protected [9]. However, it is important to note that most commercial organic coatings are permeable to oxygen and water and therefore the coatings do not simply act as a barrier [10].

It is suggested in [10] that those organic coatings protect the substrate by protecting the oxide layer that is formed on any newly exposed metal surface. Often this oxide layer is removed through dissolution into an aggressive environment, such as that present in aqueous corrosion, allowing further fresh attack. If a paint coating is present, dissolution of the oxide layer is prevented, and a level of protection is provided attributable to the decreased ease of electron transfer through the oxide layer. Hence, as long as the paint integrity is maintained the oxide layer formed will provide corrosion protection [10]. The ability of the paint to maintain this protection depends on the components and coating layers used in the coating system.

## 3. Coatings Compositions

Whilst coating of pure metals onto a substrate does still occur, more commonly alloys of a number of metals, specifically chosen for their combined properties, are used. A wide range of alloys exist for the coating of metallic substrates and their applications vary depending on the intended use of the product. Examples of metals include zinc, aluminium magnesium and chromium.

In comparison to metallic coatings, organic coatings (paint) are a more complex chemical mix of a variety of components of specific properties, each of which facilitate certain paint functions. The most common components present in paints are the resin (binder), solvent (carrier), pigments, filler and additives [9,10,11] which are described in Table 1. However, these are not a requirement of all paint systems, and it is possible one or more of these may be absent in a coating for a number of reasons.

**Figure 1:**
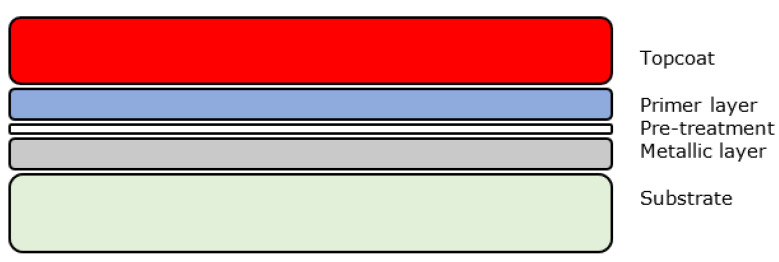
Generic organic coating layers.

Creating a single organic layer with the required properties is not usually possible and hence manufacturers use multiple layers of different composition coatings each optimised for a specific function or task. A basic system is usually composed of a pre-treatment layer, primer layer and a topcoat layer as shown below in Figure 1. For some applications a clear coat is applied onto the topcoat.

Most organically coated products also include coatings on the reverse of the substrate for aesthetic reasons and further protection. Generally speaking, however, these are either identical to the front face or only include pre-treatment and a single backing coat due to the decreased environmental requirements [13]. The properties of each coating layer are discussed in Table 2.

## 4. Coating Failure and Corrosion

For a coating to be effective it must perform without failure for the intended lifespan as there are large financial costs of paint failure due to cost of repair, loss of time and repainting [18]. Due to the complex chemistries involved in coating systems and the complexity of the environments they are placed in, the subject of coating failure and performance analysis is vast. It has, however, been reported that coating failure and damage generally follows the following sequence [8]:

1. Formation of defect—defects may be present because of poor preparation, for example lack of sufficient cleaning can leave dirt or grease on the substrate pre-coating. Alternatively, they may be formed as a result of mechanical action or degradation by, for example, temperature or UV light exposure. Finally, chemical defects may form because of reactions with acids or other chemical species that the coating may be exposed to in some environments.
2. Oxygen and ion uptake from surrounding environment—this process is dependent on the pore space of a coating which defines the amount of space between the molecular chains of a coating. The permeability of the coating, which may change with the increased presence of defects, will also determine the ease and rate of this step.
3. Coating adhesion loss—the coating begins to de-adhere from either the substrate or another coating layer. This may occur through the formation of a blister or may involve the delamination of large sections of the coating.
4. Electrolyte penetration to substrate surface—in some environments, electrolytes may be able to penetrate through the, now significant, defects in the coating. This can lead to increased adhesion loss.
5. Corrosion initiation on substrate—if electrolyte penetration occurs then corrosion is very likely to initiate. However, even though the coating is no longer intact, ion transport is still reduced by its presence. This can, hence, prevent corrosion somewhat by reducing the availability of charge balancing ions reaching the active corroding site. Unfortunately, this effect can also increase the rate of corrosion through the retention of ions that are aggressive such as chloride by restricting their diffusion away from active sites.

Defining the point of coating failure is difficult as it is widely accepted that all paints will deteriorate over time and, under exposure to aggressive environments, lose protective and aesthetic qualities leading to a perceived ‘failure’ [19]. Failures can be caused by a number of factors, shown in Table 3.

Often, however, corrosion occurs before the coating is fully degraded, this is known as under film corrosion and causes significant damage to coatings and provides the largest requirement for coating repair on constructed metal [10]. The most commonly seen under film corrosion phenomena are shown briefly in Table 4.

## 5. Techniques for Corrosion Monitoring

Table 5 introduces a number of reported techniques for corrosion and or coating monitoring that will be explored in this report.

## 6. Non-Destructive Techniques

### 6.1. Acoustic Emission

**Figure 2:**
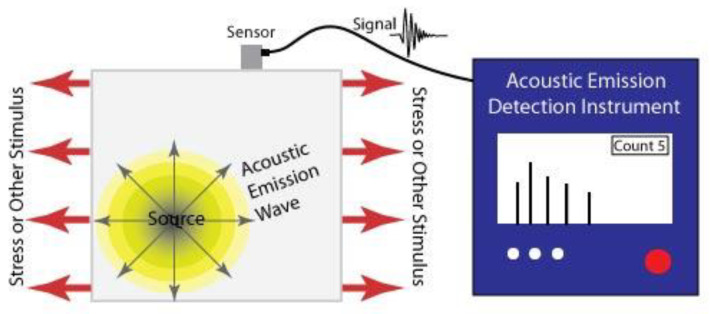
Acoustic emission detection. Reprinted by permission from, ©Iowa State University Center for Nondestructive Evaluation (CNDE) ref. [26].

The basic principle of this technique is to determine the condition of a structure by detecting sound waves emitted from it due to the sudden redistribution of stress and associating this with damage or defects [23]. Usually, cracks are the defect responsible for the generation of the sound detected and the waves generated are often dependant on the geometry of the crack [24]. Hence, this is a so called ‘passive method’, the sensor simply tells you when the sample fails in some way rather than how it is performing [25]. The basic working principle of this technique is shown in Figure 2.

Generally, the sensors are piezoelectric and are connected to the surface of the test material. They produce signals which can be easily amplified, filtered and computationally analysed [24,27]. It is important that the frequency spectrum of the sensor corresponds to the test medium, the scale of the sample and the expected defect that will be generated. Normally higher frequencies are expected from smaller specimens and vice versa [24].

A benefit of this technique is that it allows a cumulative calculation of defects and damage and hence an estimate of the remaining lifetime until failure. Use of multiple detectors can allow spatial determination of a defect location and hence propagation and severity indications [25]. It has also been reported that it can distinguish between flaws that are active (are increasing in size/severity) and those that are not [23].

A disadvantage of this technique is the required sensitivity. For example, if the sample examined is very small the acoustic emission will be of a similarly small magnitude, so detection may be difficult. There is also high possibility of noise from the surround and a difficulty in determining the size of the defect without further testing using additional techniques.

Perhaps the simplest application of this method is in determining the damage to bridge cables by measuring the sound waves generated when individual wires break. The number of emissions and the frequency of their detection can be easily corresponded to the lifetime of the cable [28].

There has been some success in using this technique for coating monitoring, most namely analysis of thermal barrier coatings when subjected to thermal cycling [27]. In this work [29] cracking of an approximately 100 μm thick coating due to thermal fluctuation was detected and an estimation of coating condition was possible. However, it is considered that while this specific application, in which the most likely coating failure method is via cracking, is useful the technique may be less applicable to detecting failure from general weathering of samples.

### 6.2. Ultrasonic

This technique utilises ultrasonic sound waves. Sound waves are the propagation of mechanical or elastic energy through materials via the mechanical vibration of particles about a fixed point [25,30].

Ultrasonic waves refer to high frequency soundwaves of 20,000 Hz and higher, representing the highest of the three regions of the sound spectrum: the others being audio and infrasonic [30]. The short wavelength associated with high frequency ultrasonic waves gives better resolution than, for example, infrasonic waves which are used for the detection of earthquakes [30].

**Figure 3:**
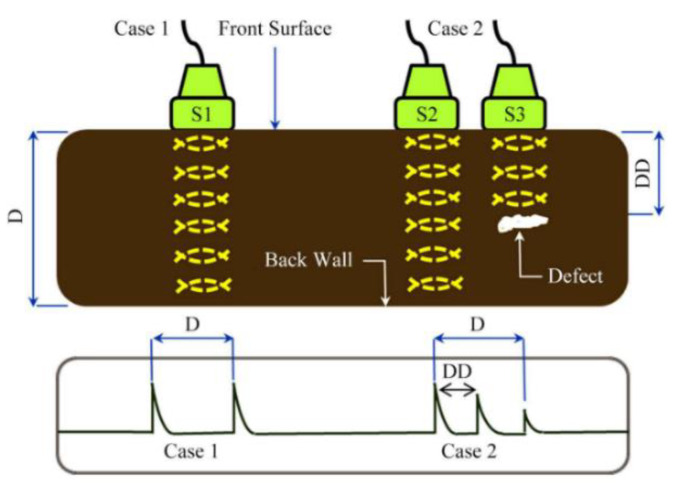
Ultrasonic testing principle. Reprinted from ref. [31].

This non-destructive technique uses the time difference between ultrasonic vibration generation and detection in order to allow accurate distance measurements to be made. Frequencies between 1 and 6 MHz are common testing frequencies used [30] and the probe is often piezoelectric. When ultrasonic waves passing through a medium encounter a different medium they are partially or totally reflected; this is due to their short wavelength, not dissimilar to the wavelength of light [30]. These reflections are picked up by a detector and a simple calculation is used to determine the distance travelled by the vibration and hence the location of defects can be determined. The basic principle is shown in Figure 3.

There are two main methods for ultrasonic testing, straight and angled beam. In straight beam the ultrasonic vibration is not continuous, and the vibration is directed in a straight beam into the sample. This is effective at detecting defects parallel to the surface of the sample. Angled beam testing is more common in weld analysis and uses vibrations at an angle to the surface to allow better detection of defects that are not parallel to the surface. This makes angled beam a more complex, but often more accurate, method [31].

Currently, the technology exists for easy affordable and reliable measurement of coatings of several mm in thickness [32]. This technique is already extensively used in the oil and gas industry for a variety of testing including measuring pipe thickness and condition of welds [31].

Unfortunately, the quality of results is affected by surface condition and dirty or rough surfaces will produce a significant quantity of noise and hence reduced likelihood to easy detection [33]. Limitations in detection due to noise are best avoided when the medium tested is homogenous in nature due to a reduction in the presence of reflections due to porosity or inclusions [24].

It is known that the accuracy of this technique is directly corresponding to the speed of sound in the measured medium [32]. Testing is also required in order to determine accurate quantitative measurements: a test piece of known thickness should be used as a reference. However, an advantage of this method is that temperature is not usually a significant variable [30].

**Figure 4:**
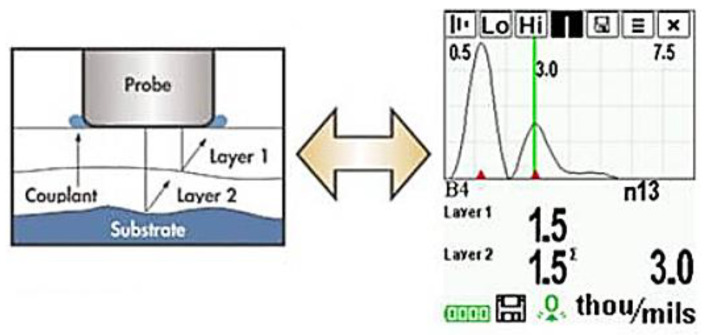
Application of ultrasonic testing to coating layers. (Adapted with permission from ref. [32]. Copyright 2004 DeFelsko).

A probe that could generate and detect ultrasonic vibrations could determine thickness measurements of coatings. It is possible that, with sufficient calibration, the determination could be made between different coating layers, as shown in Figure 4 [32]. It is important, however, to note that as these layers are decreased in thickness limitations in distinguishing layers occur due to noise and that these layers require distinct properties for accurate detection.

The potential of this technique cannot be understated as there is a great deal of information that could be determined by such testing. For example, by carrying out thickness measurements over a set time period, the rate of paint degradation could be measured, and the expected lifetime estimated. Furthermore, it may be possible to detect otherwise difficult to see defects, such as individual layer delamination or osmotic blistering by comparing the expected thicknesses of each layer to measured. Finally, the fact that this is a spatially resolvable procedure is very important; flaws are not only detected but their location and size can precisely be measured [25].

**Figure 5:**
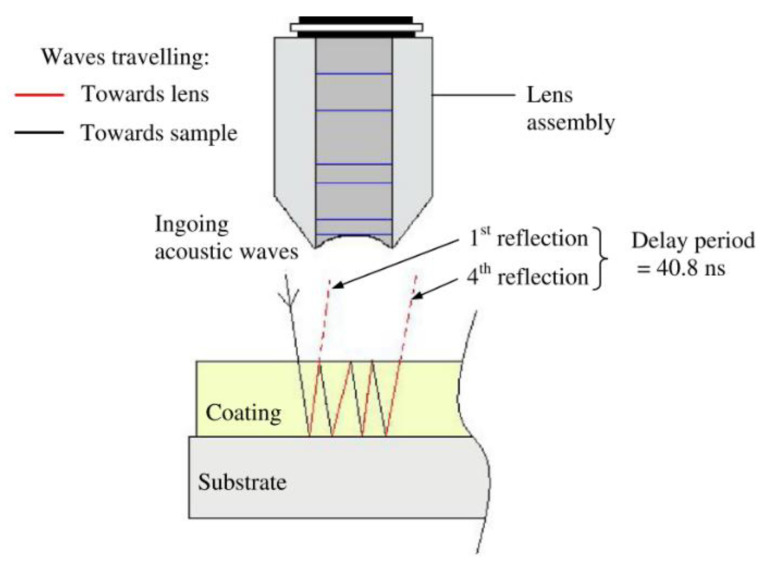
The use of acoustic waves to measure coating thickness. (Reprinted with permission from ref. [34]. Copyright 2008 Elsevier).

There has been success using other waves such as acoustic waves in the same way [34]. An example looked at lacquers on food beverage cans with the aim of detecting blisters and deformation of the coating [34]. An example of the equipment used, and the measurement method is shown in Figure 5.

However, there is concern that at the thickness that is required for some coating systems this method becomes far too sensitive to noise for in situ measurements. Furthermore, the requirement for a clean sample that is easy to access to scan a probe may limit feasibility. There is also an argument that this type of testing is too reliant on human involvement as the scanning of the probe would be difficult to automate across a complex building geometry.

### 6.3. Terahertz Waves

This is a technique similar to that of ultrasonic testing but that involves using terahertz waves, which are electromagnetic waves with a frequency between that of light and radio waves [35]. By measuring the time delay of returning reflected waves the thickness of the sample can be measured accurately if the refractive index is known [35]. Photoconductive antennas can and have been used for both detection and generation of terahertz waves and thickness measurements of 300 μm have been achieved [35].

This technique has been successfully used to measure the thickness of ceramic topcoats used in thermal coatings as it is better than ultrasonic testing at analysing thicker coatings which are more defect prone [35]. There has also been success in measuring the thickness of marine protective coatings as described in [36].

Further benefits of this technique are that it is better for analysing coatings that are opaque or that heavily scatter visible or infra-red light, when compared to ultrasonic [36]. Changes in thickness, refractive index or absorption coefficient can be detected [36] all of which may be affected by degradation of a coating or corrosion.

### 6.4. Magnetic Flux Leakage

This is the most common, non-destructive, electromagnetic technique for determining metal loss and damage due to corrosion [37]. It relies on the basic principle that the direction and strength of a magnetic field surrounding a magnet can be defined by magnetic flux lines [37].

**Figure 6:**
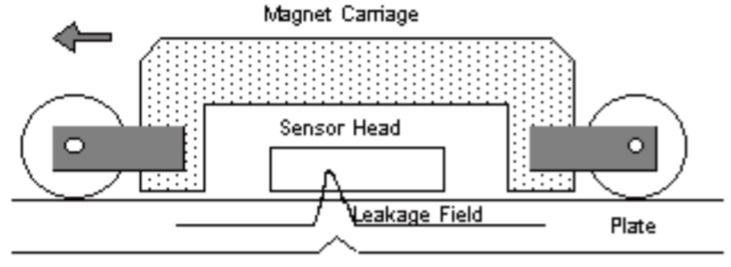
Example magnetic flux leakage device. (Reprinted from ref. [33]).

In this technique a magnet is used to saturate an area of a sample with magnetic flux. A sensor then measures deviations in the expected flux lines of the magnet; these deviations can be attributed to defects in a sample which cause the flux to ‘leak’ out of the sample due to the change in relative magnetic permeability. An example of a standard set up and the working principle is shown in Figure 6 [33].

There are two main types of sensors used for the detection of leakage field, these are Hall effect devices and coils [33]. Both produce a voltage dependent on the strength of the magnetic field passed through them based on the principles of Faradays law of induction.

This technique is used extensively in the pipeline industry where ‘pigs’, cylindrical units composed of magnetic flux leakage sensors, are sent down the inside of pipes frequently with a 90–95% detection rate for defects greater than 10% of the pipe thickness [37].

When used on clean surfaces up to 10 mm there is reliable detection of 10–20% thickness changes and scan speeds of 0.5 m/s are possible [33], however, the cleanliness of the sample is crucial in reducing noise and hence samples are often cleaned before measurement.

It should be noted that MFL can still maintain sensitivity when the test sample is surface coated [33] and hence is expected to be able to give useful data of the condition of the substrate and hence the effectiveness of the coating. This is proven in [38] where a MFL device is used to detect artificial defect cracks down to 1.4 mm in a steel sheet even when various composition coatings up to 30 mm are used. Although organic coatings were not used in this work it is expected to behave similarly.

However, it is evident that this technique is limited to ferrous materials for obvious reasons and calibration to the specific magnetic permeability of the specimen is also required [33].

A variation of magnetic flux leakage, known as magnetic particle testing (MPT), uses a magnetic powder to visually detect the defects. The magnetic powder is attracted to the flaw regions as where ‘escaped’ magnetic flux is strongest and hence indicate the presence of flaws. Despite common use in NDT testing, it is unlikely to be easily automated as a method of testing due to complications in cleaning and applying the test medium [39].

### 6.5. Magnetic Adaptive Testing

This is a fairly newly developed technique which is based upon the principle that structural defects in a ferromagnetic material will affect the magnetisation of that sample [40]. It has been suggested that different flaws will have unique effects on the magnetisation process [40]. The magnetisation of an object is often considered by examining the hysteresis loop, the relationship between the magnetisation of a material with an applied magnetic field intensity. Hence, it is expected that, by analysing a large number of subsequent hysteresis loops for a sample, the health of the sample can be calculated. This is done by measuring the voltage induced in a pickup coil as the magnetising field changes [40].

It is accepted that a large amount of experimentation will be required in order to determine the type of defect or to be able to determine severity. This method has been suggested for use in nuclear reactor pressurised containers, however, fatigue testing of samples is also thought to be an application.

The applicability of this technique is limited as it has mainly been applied to measuring strain in metal samples, which may be difficult to relate to corrosion of the sample. Furthermore, measuring the magnetic properties of the material in situ would be complex.

### 6.6. Magnetic Memory Method

This method developed by A.A Dubov determines stress concentration zones by detecting changes in the residual magnetisation field [41]. This has been described as ‘the measurement of self-magnetic flux leakage’ (SMFL) [42]. It relies on the detection of abnormal magnetic fields without magnetisation or stimulation. These are developed as a result of stress concentration zones and hence allow predictions of lifetime of a sample [42].

Stress concentration zones accumulate as irreversible changes in the magnetic domain of structures under load in ambient geomagnetic conditions. This means that the magnetic state of a sample at any point is related to the concentration of stress through structural damage. Hence, measurement of SMFL and its distribution can give information about structural health [42]. These measurements are made using a magnetometer which detects ‘places of maximum inhomogeneity’ shown by describing changes in magnetic field components at any point [41].

The disadvantage of this method is that its practicality outside a theoretical assessment has yet to be proved and therefore it may not be as accurate or quantitative as other techniques [42]. Furthermore, this technique has only currently been applied in any detail to pressure pipes and pipelines [42]. The lack of quantitative data received implies that this technology can only accurately be used to determine points of interest for further testing and there is difficulty in relating measurements to the actual level of damage present [42].

However, it does have the benefits of early failure detection and ease of use and it does not necessarily require application of an external magnetic field. With sufficiently sensitive measurement sensors ambient magnetic fields can be used instead leading to less equipment demand than, for example, magnetic flux leakage. Other benefits include good resolution, up to mm level, that can be measured at a speed in the order of meters a second [42].

### 6.7. Eddy Current Testing

**Figure 7:**
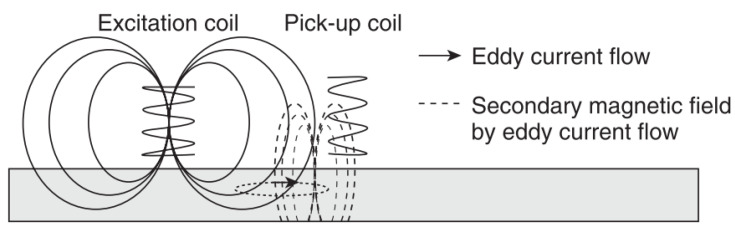
The principles of eddy current testing. (Reprinted with permission from ref. [45]. Copyright 2013 Elsevier).

This is a form of electromagnetic testing based on the principle of electromagnetic induction [43,44]. By passing an alternating current through a coil a magnetic field is produced and this in turn produces eddy currents in a sample when placed in close proximity to it; these eddy currents then produce their own magnetic field which opposes the primary field. Defects cause a change in these secondary magnetic fields and hence produce a shift of impedance on the primary coil [45]. These impedance changes or simply changes in the secondary magnetic field are measured [45,46]. Hence, changes in conductivity and permeability are measured and these can be related to corrosion effects [45]. Frequencies between 50 Hz and 1 MHz are commonly used [43] and an example of this principle is shown in Figure 7.

**Figure 8:**
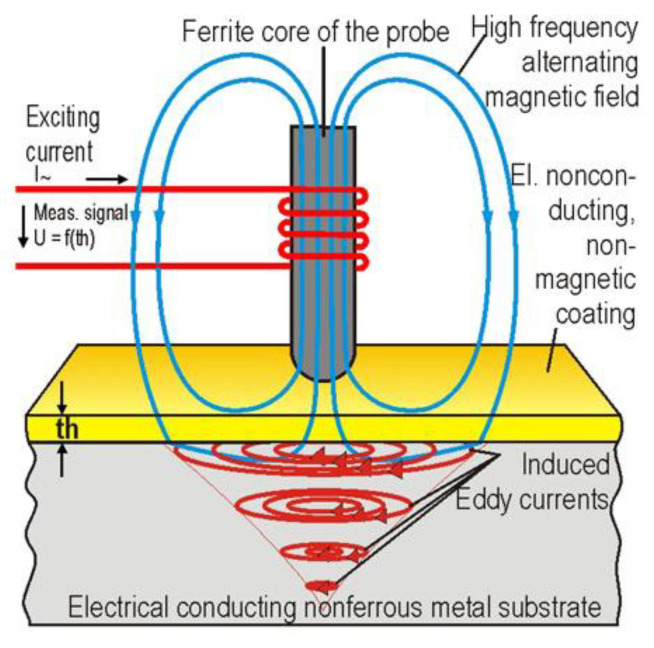
Use of eddy current testing on organic coatings. (Adapted from ref. [48]).

Although often used for metal substrates it can also be used to measure organic layer thicknesses as shown in Figure 8. This is possible by monitoring the feedback effect produced when the probe is placed near a coated substrate as described in [47].

Advantages of this technique include good sensitivity and the ability to penetrate through layers on top of the substrate. These could be coating layers or accumulated dirt which means that pre-cleaning of the substrate is not as crucial as with other testing, such as magnetic flux leakage [43]. It has also been reported that coatings of up to 5 mm thickness can be penetrated which is far thicker than the coatings for the scope of this work [43]. There are also benefits of easier automation and portability when compared to other non-destructive techniques [43] such as ultrasonic. It has also been suggested that under coating corrosion (of few mm) can be detected in early stage, independent of thickness of coating (300–1000 µm) or presence of corrosion product [46].

Disadvantages include accuracy issues, caused by natural variation of permeability values of the substrate and coating, and the complexity and difficulty in signal interpretation [43]. Furthermore, it has been suggested that defects out of the plane of view are often not detected and those scans of geometries that are complex is difficult to implement [43].

Insulating coatings for storage tanks have been analysed using eddy current testing with success. These were an epoxy glass reinforced phenolic coating and a modified epoxy on steel substrate as documented in [46]. There has also been some success using eddy currents to measure the integrity of thermal barrier coatings on gas turbine blades [49]. Example of developed sensors such as [47] exist as do the use of eddy current testing for measurement of non-conductive coatings such as [44,50]. Heterogeneities in the coating can lead to a phenomenon ‘lift-off variation’ [46] and this lift off effect can be used for thickness measurements for thicknesses of 0.5–25 μm

Furthermore, a technique called ECCPC (eddy current co planar coil configuration) has been developed for measuring thickness of non-conducting layers and is documented in [50]. However, summarily to ultrasonic testing these devices suffer in terms of ease of automation, scanning complex large geometries with human interaction may be difficult.

### 6.8. Infrared Thermography

This is a technology that relies on the principle that the thermal signature produced by a component will be dependent on the presence and severity of defects in that sample [51]. There are two types of infrared thermography: active and passive. In active thermography the sample is stimulated with a fixed quantity of infrared energy and the resulting thermal signature, when the infrared source has been removed, is measured. In passive thermography the sample is not stimulated with any infrared energy, the ambient thermal signature is simply monitored [52].

It is generally agreed that using active thermography allows much better characterisation and detection of defects [52]. Hence, a commonly used experimental set up will be composed of a heat source and a detector [51]. Often the detector is simply a sensitive infrared camera, however, a variety of heat sources can be used from a simple heat gun to devices using electromagnetic radiation and electromagnetic effects to heat the sample [51,52].

Advantages of this technique are that it can be used on any material, but effectiveness does depend on factors such as the thickness and the material thermal properties [51]. It can also be used to analyse a large area rapidly on a variety of sample shapes [51] and no contact to the sample is required so it is often easier to implement than another non-destructive testing method [51]. Finally, the equipment is relatively safe and simple [52].

The main disadvantage of this technology is that it requires very sensitive infrared sensors. It is also of crucial importance that the stimulating infrared source supplies an even reproducible quantity of energy to the sample. Due to the sensitive nature of this method, external factors present in situ, such as sunlight and other weather features, could drastically reduce the accuracy of measurement.

**Figure 9:**
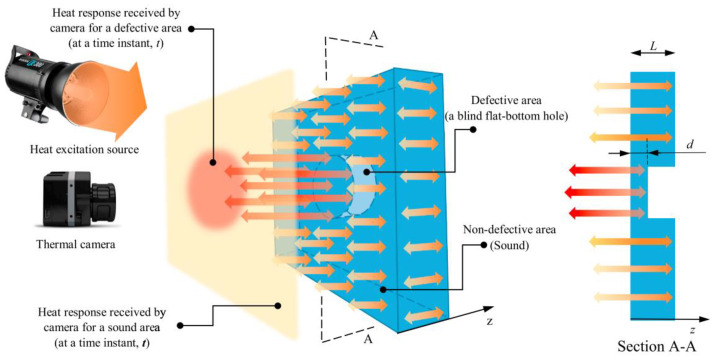
The principles of thermography (Reprinted with permission from ref. [52]. Copyright 2019 Elsevier).

There has been some success of detecting corrosion under coating, blisters, ruptured blisters and filliform [53]. There is also some evidence to suggest that this technology can allow the size and severity of the corrosion to be determined [53]. An example of active thermography operation is shown in Figure 9 below [52].

### 6.9. Pulsed Thermal NDT

This is a form of thermography testing in which the sample is thermally stimulated using a series of pulses of thermal energy and the resulting thermal field is analysed [54]. Stimulation can be through a variety of methods including mechanical waves, optical radiation, microwaves, inductive methods, electrical or eddy currents or through heated gasses [54]. As the stimulating source can be more finely controlled, temperature deviations should theoretically only occur at areas where there are defects [54]. The benefit of this specific version of thermography is that it can be used over large areas, using a linear or on a point-by-point test basis, and produces spatial determination of defects. For all stimulation methods the distance between the sample and source/detector are important factors in the overall sensitivity of the method [54].

### 6.10. Eddy Current Pulsed Thermography

**Figure 10:**
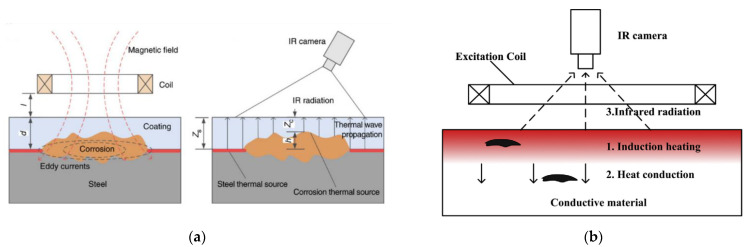
The principle of eddy current thermography for use in (**a**) corrosion detection and (**b**) generic defect detection. (Reprinted with permission from ref. [55,56]. Copyright 2017 and 2014 Elsevier).

This is a variation of thermography technology that has been used in [55,56] to detect corrosion blisters in coated substrates. It involves heating a substrate through a coating using electromagnetic induction and analysing the resulting thermal patterns created [55]. Hence two steps are involved: through coating heating and through coating imaging [55]. Eddy currents that are generated in the substrate, by application of a magnetic field, generate heat through resistive heating and joule heating [56]. This heat then conducts through the material until there is a thermal balance set up and infrared radiation is then emitted from the sample [56]. An example of this process is shown in Figure 10.

**Figure 11:**
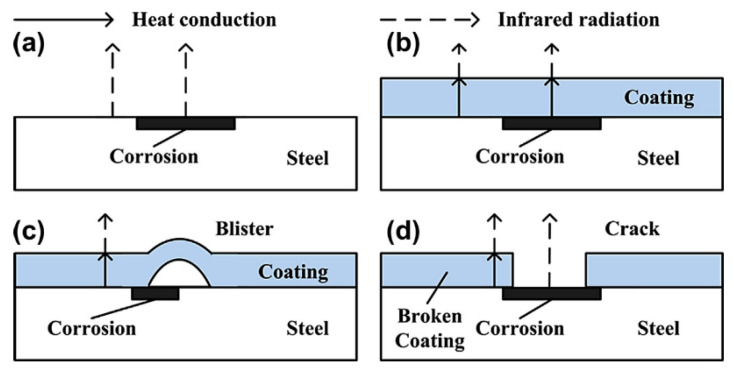
The effect on the measured thermal energy of different coating failure modes: (**a**) corrosion, (**b**) under coating corrosion, (**c**) blistering, (**d**) coating failure. (Reprinted with permission from ref. [56]. Copyright 2014 Elsevier).

At areas where the coating is deteriorated a hot spot is detected due to the reduced insulating effect given by a deteriorated or thinner coating. Experimentation has shown that corrosion on a non-coated sample shows as a hot spot, as does corrosion under a coating although this effect is reduced by the coating present. A blister in a coating will show as a cold spot due to the increased distance and material that the infrared radiation has to penetrate to be detected. As expected, a crack or broken coating will show as a hot spot [56]. This is displayed in Figure 11.

This technology combines the benefits of eddy current testing with the large scale, rapid imaging of thermograms [55]. Non-uniform heating is the main barrier to accurate measurements with this technology. The sensitivity previously mentioned is also a potential limitation of this method and it requires experimentation on each coating used in order to determine the thermal barrier contribution of different compositions [56].

## 7. Optical Techniques

### 7.1. Corrosion Indicating Paint

This works on the principle that there is some component present in the paint layer which can signal the onset of corrosion by way of some reaction with a component of corrosion.

There has been some success with redox and metal ion complex fluorescent materials; these either become fluorescent when oxidised (redox) or fluoresce as a result of forming complexes with metal ions produced through corrosion (metal ion). An example is Oxine which fluoresces when reacted with corroded aluminium, or Fluorescein [57].

**Figure 12:**
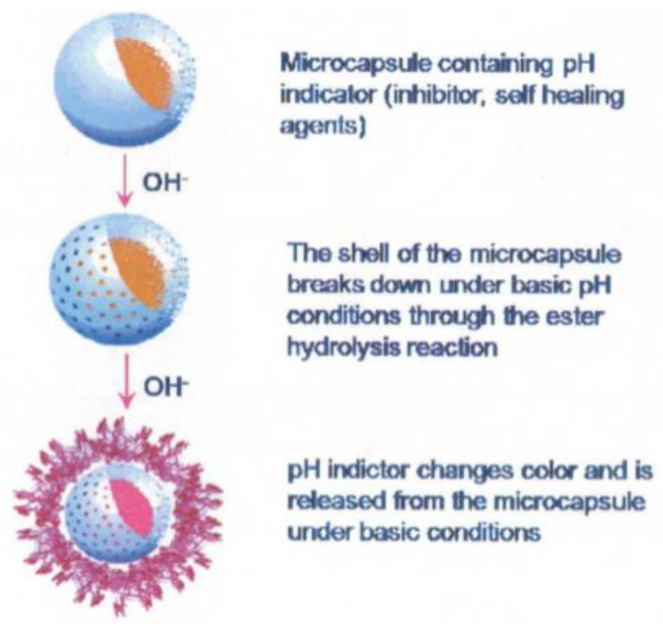
The principle of a smart release pH triggered microcapsule (Reprinted from [59]).

Another approach involves detecting changes in pH as, in localised corrosion, it has been shown that there can be large changes in acidity or alkalinity. pH triggered microcapsules can be made from 1 micron in diameter upwards with a release time of approximately 4 h in pH 8–10 or 1–4. An example of these microcapsules is shown in Figure 12. They can either contain dyes to indicate where this localised corrosion is occurring or healing agents to arrest the corrosion. Current work includes making the paint itself a fluorescent dye capable of responding to pH changes [58].

**Figure 13:**
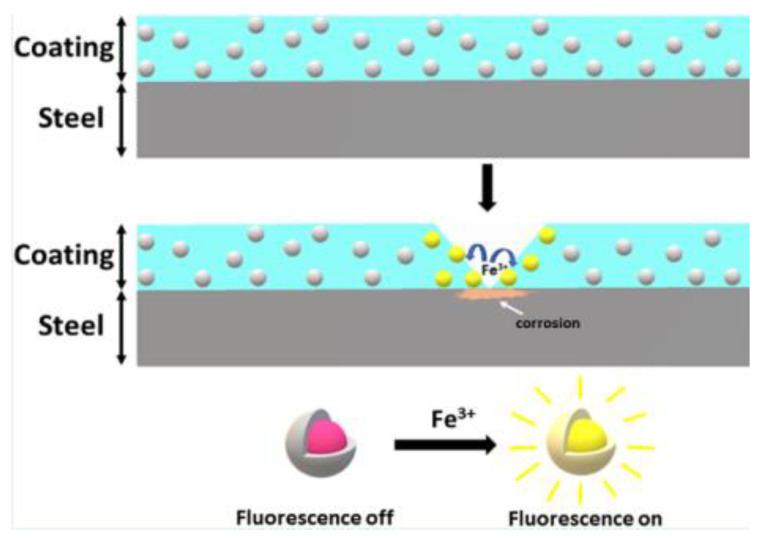
The principle of corrosion triggered fluorescent microcapsules. (Reprinted from ref. [60]).

A recent example of this approach is given in [60] which describes the development of a nano-sensor composed of dispersed mesoporous silica shells filled with organic molecules. These molecules undergo a reaction with iron ions present due to metal dissolution during corrosion causing fluorescence as shown in Figure 13. Alternatively the shell of these capsules can be composed of ester or thioester groups which undergo hydrolysis under high pH conditions releasing the indicator [58]. It was reported that these particles allowed corrosion detection before visual detection of corrosion products (iron oxide) and has also been successfully implemented in an organic coating. Measurement of the fluorescence was carried out with a fluorescent microscope and FCS (fluorescent correlation spectroscopy) [60].

Although using a dye directly would be easier to implement, by using microcapsules issues such as dye solubility in the paint system can be avoided [58]. It has been reported that oil core capsules were compatible with water-based paints and solvent based paint compatible with water core capsules [61]. Furthermore, adhesion testing and basic corrosion testing has shown that there was little effect of the addition of these microcapsules on the coatings’ properties [61].

The advantages of this technology are that, theoretically, the capsules can contain anything including liquids and solids. Hence a wide range of indicators may be used, or the capsules could facilitate implementation of new inhibitor systems without having to modify paint characteristics. Furthermore, as the mechanism of release does not require mechanical breaking these capsules can be used to detect or combat corrosion under a film [61].

A potential disadvantage is that the microcapsules sit in the paint system and could potentially interfere with the adhesion or protective qualities although some testing by Li et al. has suggested this is limited for certain coatings [58]. However, the potential of capsules to rupture during the coating process or to disrupt current methods of painting and curing are as yet not fully documented. It may well be the case that these capsules may be unsuitable for current coating methods leading to decreased ease of implementation. Furthermore, it may be considered undesirable to have visual indicators of corrosion as it could lead to unsightly patches of colour change on a façade.

### 7.2. Stressed Optical Fibres

**Figure 14:**
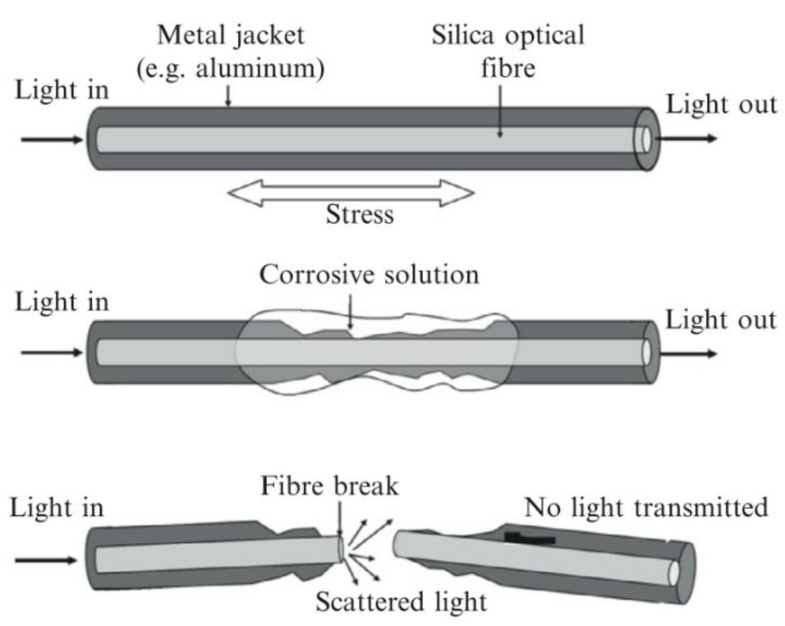
The basic principle of a corrosion fuse. (Reprinted with permission from ref. [62,63]. Copyright 2008 and 2014 Elsevier).

In the literature these are also referred to as a corrosion fuse [62]. This technology involves embedding stressed glass optical fibres in the medium to be monitored. As the medium is corroded or begins to fail the stressed optical fibre is gradually exposed until the medium no longer supports the fibre and it fails. By passing light through the fibre and measuring the intensity, it can be determined when the fibre has failed [62,63]. A visual description of this process is shown in Figure 14 [62,63].

By embedding fibres at different depths, there is the potential that the rate of penetration can be calculated, and a lifetime estimate, or maintenance schedule could be determined. Alternatively, by having several optical fibres coated in differing quantities of metal a rate of corrosion could be estimated.

There has been some evidence suggesting that aluminium coated fibres under testing show results that correlate with expected corrosion times [62] suggesting the technique provides accurate results. There are also some commercially available wire sensors (Cosasco) which allow monitoring by passing a current through them reducing the requirement to use thin brittle glass fibres.

Fan [64] used optical fibres to measure the expansion due to corrosion of steel fibres in reinforced concrete. In this case BOTDA (Brillouin optical time domain analysis) was used to measure the strain imparted on the optical fibres. It was reported that strains as small as 100µε could be recorded [64].

The advantage of this technique is that is it very easy to analyse the data produced and directly relate that to the coating condition. A potential disadvantage of this technology is that it may, itself, reduce the coating protective properties by decreasing adhesion or increasing the likelihood of defects forming. Additionally, it is difficult to imagine how this could be scaled up to factory levels of production considering the fibres are extremely brittle and thin. Depending on the coating thickness there may also be aesthetic issues created by using embedded fibres and there are limitations to how thin the fibres can be made and handled.

### 7.3. Bragg Gratings

A Fibre Bragg Grating (FBG) is an optical fibre that has periodic changes in refractive index along certain lengths of their fibre. This has the effect of creating an optical filter that reflects certain wavelengths and only transmits others [63]. The effect of this is that any changes in pitch length of the fibre can be measured by relating these variables to back reflection at certain wavelengths [63]. This is given by the formula:

(1) $$\lambda B=2n_{eff}\Lambda$$

where *λB* is the resonant wavelength, *n_eff_* effective refractive index, *Λ* pitch length of the grating [63].

**Figure 15:**
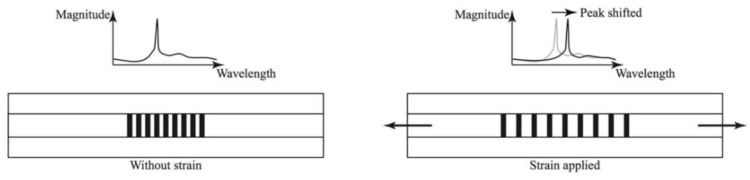
The response of a Bragg grating to an applied strain. (Reprinted from ref. [66]).

These changes could be induced by deformation, induced by corrosion, or could be down to a coating failure type that involves deformation of the coating; for example, osmotic blistering. Therefore these sensors could potentially be used to measure and determine corrosion related strains as well as coating issues, such as delamination [65]. As changes in strain occur in the coating, due to corrosion or failure, the Braggs grating wavelength changes and the corrosion rate can be calculated [65]. The effect of strain on a Braggs grating is shown in Figure 15.

**Figure 16:**
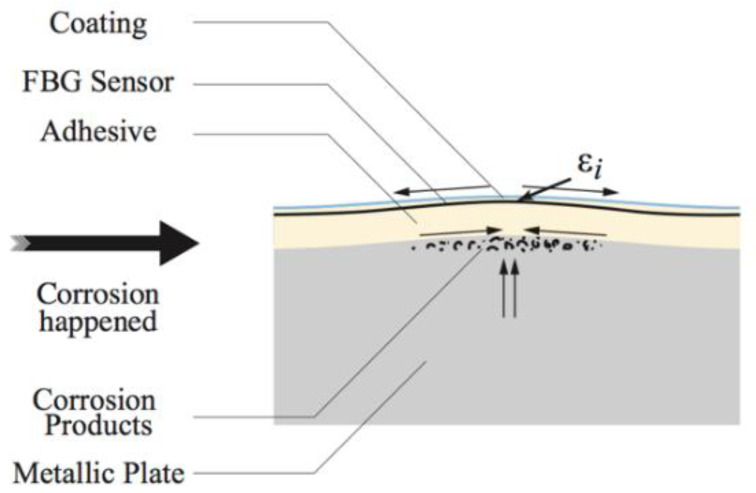
Detection of corrosion blister forming under a coating by a Braggs grating (Reprinted from ref. [65]).

Many companies have developed this technology and there has been some implementation success in the civil industry using integrated FBGs to monitor static building structures [63]. There has also been some success in embedding these sensors into coatings (polymeric and metallic) on top of steel [65,66]. These studies showed that results from FBGs could give readings that correlated with electrochemical tests carried out simultaneously. Furthermore, FBGs have been deployed into concrete rebar structures to measure corrosion [65] and it was reported that the FBG method was within 1% of the calculated electrochemical corrosion rate [65] (for a metallic coating). An example of a Braggs grating detecting the formation of a blister in a coated product is shown in Figure 16.

Potential advantages of using this technology include the ability to determine damage to the coating as well as movement of the coating due to delamination or blistering. Their high sensitivity, low cost and ease of installation are further benefits of FBGs. It has also been shown that the Bragg fibre is capable of surviving thermal metal coating deposition so it is expected to be able to survive application of a paint system [66]. Finally as they are a well-developed sensor technique they are available commercially at high quality and it has been suggested that measurement of crack initiation and corrosion rate under polymeric coating is possible [65].

The main disadvantage of the Braggs grating is that it is affected by temperature so a temperature reference sensor is required [65]. Furthermore, the coating may fail via a method that does not induce large strains in the coating, for example UV degradation, and the effect on embedding these systems on the lifetime of the coating is not well documented.

### 7.4. Corrosion Product Detection

It has been suggested that it may be possible to optically view the product of corrosion using optical fibres [67].

One study looked at the production of Al ions when airframes begin to corrode [67]. In this test, aluminium ions were made to react with a compound to form a fluorescent metal ion complex. By passing UV light down a fibre, it was possible to detect the presence of Al ions by detecting the presence and intensity of fluorescent light. Some factors such as pH and water absorption were observed to affect the ease with which the chemistry could be controlled [67]. Although experimental and subject to complex manufacturing requirements, it offers an insight into other ways optical fibres may be used.

### 7.5. Interferometry

**Figure 17:**
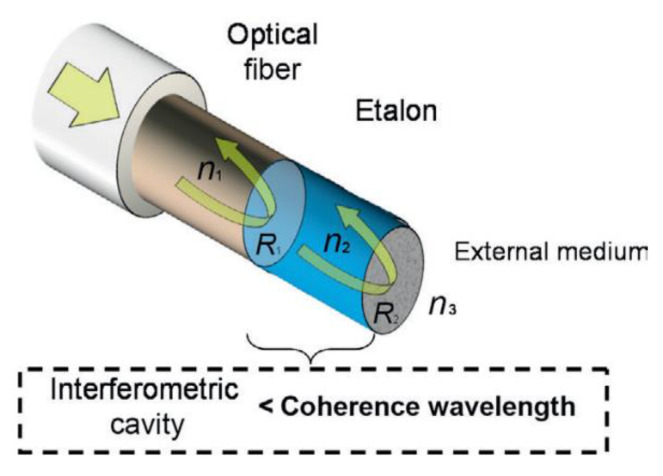
Optical fibre interferometer. (Reprinted with permission from ref. [63]. Copyright 2014 Elsevier).

This is a device that measures distances by comparing the phase change achieved between light that has reflected off the front and back interfaces of a sample. This phase change can be related to the thickness between the two interfaces of the cavity by the equation: [63]

(2) $$\delta =\frac{2\pi }{\lambda }2nl\;cos\theta$$

where *δ* is the phase shift, *λ* is the wavelength, *n* is refractive index of cavity, *l* is thickness of cavity and *θ* is angle of the light. An example of a device made of an optical fibre used to measure coating thicknesses is shown in Figure 17.

**Figure 18:**
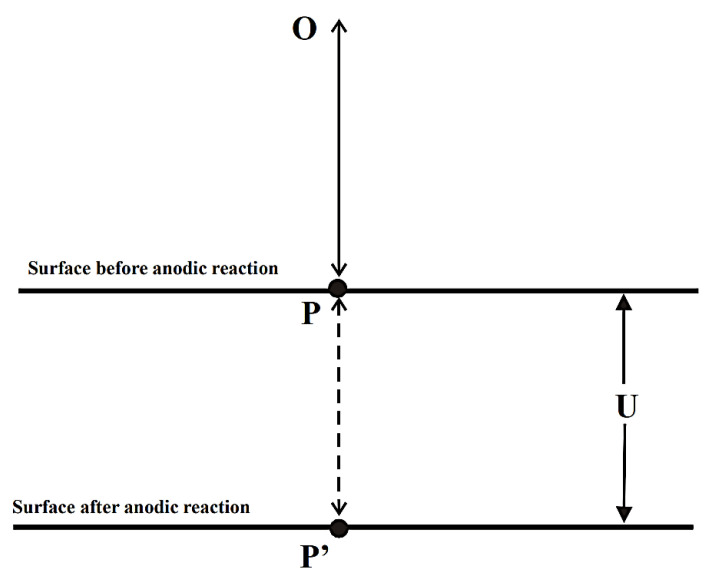
Effect of corrosion on path length of light used in interferometry. (Reprinted from ref. [68]).

These devices are used for measuring a variety of variables such as strain, pressure and displacement [63]. There has also been some success in using this technique to monitor ABS coated copper by detecting changes in distances [68] and it has been reported that 2D and 3D microscopic profiles may be constructed from testing. In this case, the difference in the sample thickness before and after corrosion was measured as shown in Figure 18.

A commonly used method, the Michelson interferometer, involves using a beam splitter and two light paths and precise measurements can be made through analysis of the interference pattern. Advantages of this method include very accurate distance measurements; disadvantages include the large quantity of equipment and the sensitivity. Furthermore, the high likelihood of noise may limit in situ suitability and poor durability that may mean the sensor is damaged during panel installation or maintenance.

## 8. Gravimetric Techniques

### Corrosion Coupons

Possibly the simplest of all the sensor technologies, these are samples that are weighed, exposed to an environment for a set amount of time and then cleaned and reweighed to calculate the rate of mass lost [69,70]. Quicker and more accurate readings can be achieved by using coupons with larger exposed surface areas and hence coupons come in a variety of shapes and sizes depending on the requirements of the situation [69].

Often coupons are used as an indicator of the corrosivity of an environment and how this varies across a building envelope. Coated corrosion coupons are used to allow measurement of paint integrity, but similarly these require manual testing or inspection at periodic intervals.

Advantages of corrosion coupons include their low cost and ease of use. They are durable, compared to some other techniques, and there are no complicated electronic control systems or power requirements [69]. They can also be used in any environment and be made out of virtually any material required [71].

The disadvantages are that they can take time to produce data and it is possible the sample does not reflect the bulk material being measured. Furthermore, readings are time consuming and prone to human error and the often localised nature of corrosion is neglected by solely testing with coupons [69]. There is also a requirement that the coupon is not interfered with by any external effects during testing [71].

Despite very common use in civil and the oil and gas industry there is little evidence that they have been applied to the determining of in situ performance of organically coated steel. The main issue with this technology is that it just shifts the item to be monitored from the building to this coupon, manual inspection and testing is still required however now the sample may be less representative.

## 9. Electrical Techniques

### 9.1. Galvanic

This is a similar technique in some respects to corrosion coupons, however, relies on the principle of galvanic corrosion. The system is composed of two metals, one which is more electronegative and hence becomes the preferential anodic site. The current of the produced galvanic cell is measured and can be directly related to the corrosivity of the environment [72]. Often measurements are taken using the ZRA method [72].

### 9.2. Zero Resistance Ammetry (ZMA)

**Figure 19:**
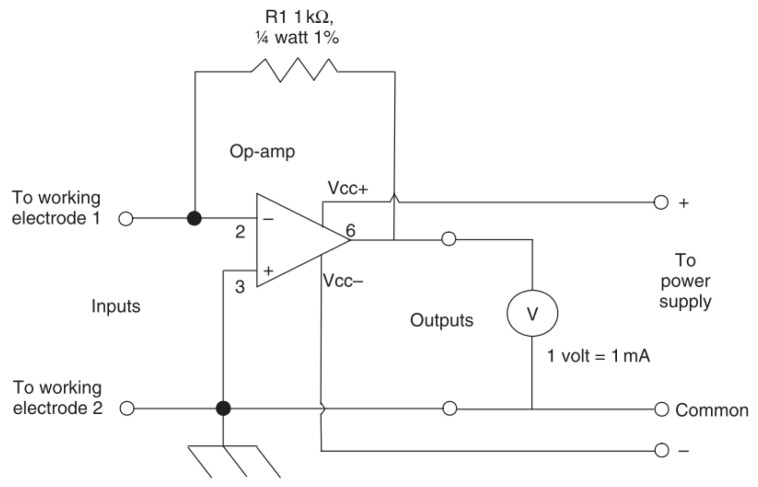
A common circuit for a zero-resistance ammeter (Reprinted with permission from ref. [73]. Copyright 2008 Elsevier).

A standard ammeter will allow the determination of a current by conversion of measured voltage across a resistor by Ohms law [73]. Zero resistance ammeters reduce the chance of interfering with the measured experiment by using a feedback loop to reduce the potential difference at the input terminals to zero [73]. The current required to do this is then converted into the voltage between the outputs [73]. A common circuit design of a zero-resistance ammeter is shown in Figure 19.

Hence, by exposing two electrodes to a medium the current required to null the resulting voltage can be measured and related to the corrosion rate of the active electrode [71].

Examples of this principle include atmospheric corrosivity measurements. In this case, by measuring the voltage between two electrodes exposed to the atmosphere a measurement of corrosivity can be made. However, it is known that these sensors usually underestimate corrosivity compared to other sensors and have been seen to observe changes in measurement even in a stable environment [73]. There has been some experimenting to develop a ZRA method of directly measuring a coated sample; this is a relatively poorly developed technology but is documented somewhat in [74].

A technique for monitoring rebar in concrete, using the ZRA approach, involves monitoring the rebar as the working electrode and the concrete as the reference, however, it was seen that unless the active and passive regions are physically separate it is difficult to determine between the two [75].

Advantages of ZRA include simplicity and low cost. It has also been shown to be effective at monitoring localised corrosion events [73], however, it does not produce as much useful data as EIS, a similar technology. It is also difficult to integrate into coatings in order to measure coating integrity and the effect of integration would have to be considered. Perhaps this technology would be useful for measuring where on the building envelope is more or less corrosive and hence where the focus of other sensors should be.

### 9.3. Electrical Resistance (ER)

This is also referred to as an electronic coupon [76]. The working principle is to monitor mass lost due to corrosion via the electrical resistance of an electrode [76]. Corrosion will cause the cross sectional area of an electrode to decrease and this decrease is measured as a resulting increase in electrical resistance of the electrode [76].

The resistance of the probe is calculated via Ohms law by measuring the voltage produced when a small current is applied to the electrode; this is compared to a reference electrode which is shielded from the environment [76].

Sensors based on this principle have been implemented in the oil and gas industry, amongst others [76]. Although it depends on the size and design of the probe, previous work has shown that the smallest change in thickness that can be detected is 1 µm.

This technique can provide information as to corrosion rate by monitoring the decrease of the thickness of the test piece [77]. Generally, it is agreed this technique is much more useful than corrosion coupons as there is no assumption of uniform corrosion rate and hence a much better indication of the corrosion rate and how this is varying is produced [76].

Advantages of this method include low maintenance, reliability and easy interpretation of data. ER sensors can also provide real time data and, compared to other technologies, are relatively easy to operate [76]. They can also be used to give corrosion rate data by calculating rate of thickness decrease [77]. Furthermore, they do not rely on the medium having certain characteristics; it can be used in any medium and gives simpler data analysis due to a lesser influence of solution resistance [77]. Finally, they can be used in aqueous and non-aqueous electrolytes, thin films and are, due to improved sensitivity, better suited for atmospheric measurements [76].

Disadvantages of ER sensors include the requirement to ensure the environmental conditions faced by the electrode are the same as the sample being studied, i.e., not shielded or fouled to a greater extent. There are also limits on the temperature and pressure at which they can be used with 100 bar and 500 °C quoted [76]. The sensitivity is often too low for rapid changes to be detected and this method also assumes uniform corrosion over the entirety of the electrode. Finally, precipitates or other fouling can form on the electrode leading to underestimates of corrosion rate [76].

**Figure 20:**
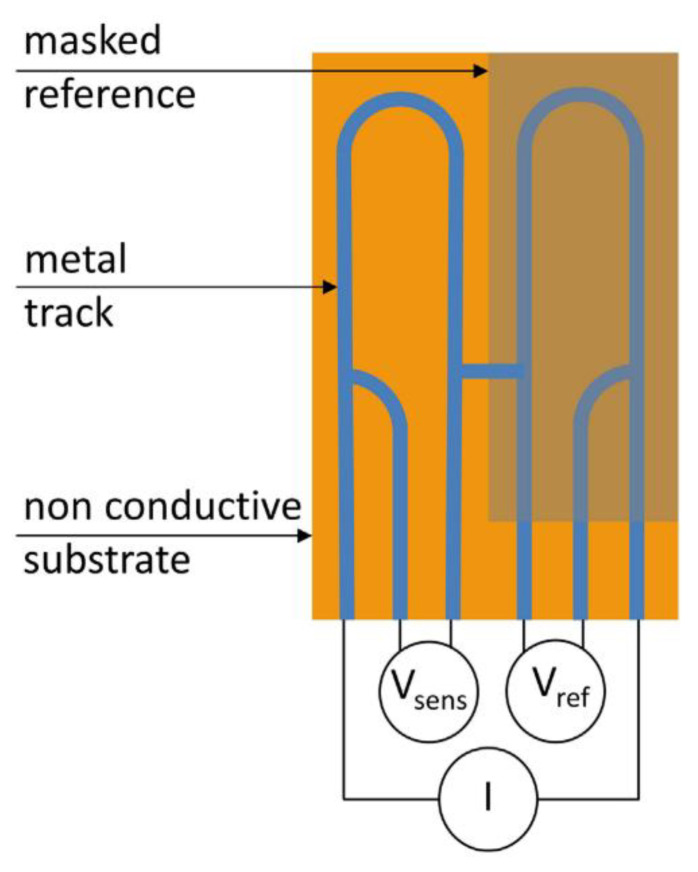
An ER probe (Reprinted with permission from ref. [78]. Copyright 2017 John Wiley and Sons)).

Diler [78] concluded that the time to failure of coating could be accurately determined using this method and that electrical resistance probes can be used with both organic and metallic coatings [78]. An example of an ER probe for organically coated samples is shown in Figure 20.

### 9.4. Induction Resistance Probes

This is a variation of the ER method in which inductive resistance is used to determine the quantity of metal loss [76]. The principle is that a decreased thickness leads to a change in the magnetic permeability of the electrode and hence mass changes can be measured by detecting changes in the inductive resistance of an internal coil [79].

Compared to standard electrical resistance probes, sensitivity is greatly increased as greater changes in magnetic permeability are seen for the same thickness change. There can also be a decrease in the response time of up to 2500 times compared to electrical resistance probes [76]. The main disadvantages of this method are that magnetic materials must be used and shorter probe lives are seen [76].

### 9.5. Capacitive Sensors

Capacitance is an indication of a capacitors ability to hold charge. Applying a voltage to two conductors separated by a medium allows negative and positive charge to accumulate on the plates [80]. If the voltage is alternated the resulting alternating current formed by the alternating charge is proportional to capacitance [80].

The advantages of capacitive sensors is that they can be used for a number of measurements such as displacement or strain [81] and they can be high resolution. It is also possible to create low power sensors that are cheap and recent development has looked at feasibility for RFID compatibility [81]. It has also been reported that these sensors are better than inductive, magnetic or eddy current methods [82] and it has also been shown by Zang [82] that coating thickness can be measured.

Capacitance can also been used to measure other features, such as the rate of water diffusion through a coating, to determine the extent to which the coating has degraded [83]. The following equation is derived:

(3) $$D=\frac{L^{2}\pi }{4t}{\left[\frac{\mathrm{lg}\left(\frac{C_{t}}{C_{o}}\right)}{\mathrm{lg}\left({\frac{C_{\infty }}{C}}_{o}\right)}\right]}^{2}$$

where *D* is the diffusion coefficient, *L* is thickness of coating, *t* is time *C* is capacitance at time (*t*), start (*o*) and fully saturated (∞) [83].

The main disadvantage of capacitive sensors is interference which causes noise and difficulty in producing accurate measurements [81]. However, it has also been shown that the penetrability of a coating is calculatable [83] and capacitive sensors have also been shown to be able to be printed inside organic coatings [84].

**Figure 21:**
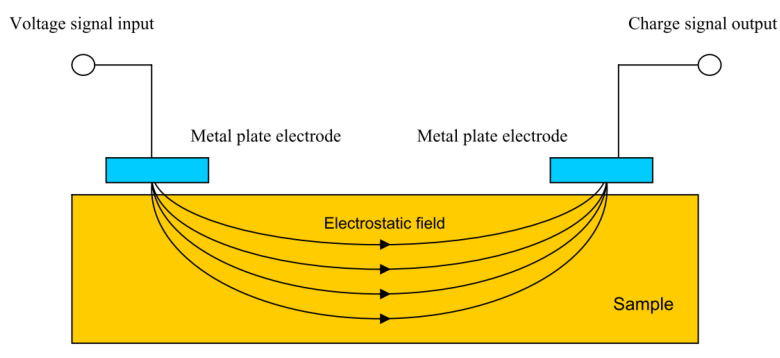
Capacitive sensing set up. (Reprinted from ref. [85]).

Capacitance theory can also be used to carry out what is known as capacitive imaging. This is a form of non-destructive testing in which two capacitor electrodes are placed near a surface a set distance away from each other, as shown in Figure 21. An electrostatic field is formed between the two electrodes which passes through the sample to be monitored.

In effect a capacitor is formed in which, by moving the electrodes, changes in substrate can be monitored. Defects in both conductive and non-conductive substrates are detectable as are changes in dielectric constants due to, for example, water absorption into a coating [85]. However, it has been stated that only surface defects may be detected in conductive specimens [86].

Compared to eddy current testing, changes in magnetic properties of the sample do not affect the measurement principles of capacitive imaging [85]. This method does, however, decrease in accuracy if the surface of the sample is contaminated or there is high moisture content. This is due to the effect of these conditions on the dielectric properties and hence may limit the feasibility to use this method in situ.

### 9.6. Electrical Field Signature Method

**Figure 22:**
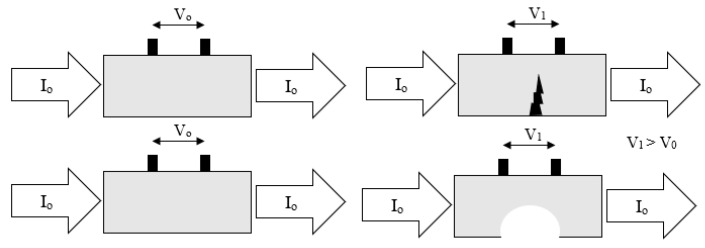
Basic principles of the FSM technique (Adapted with permission from ref. [88] Copyright 2011 Elsevier).

The basic principle of this technique involves passing a current through the test piece which is under observation and measuring the resulting potential difference field to determine the electric field pattern. By monitoring changes to this electric field pattern, corrosion information such as thickness and metal loss can be determined [87]. Measurement of the potential difference field is achieved using pick-up pins which are permanently installed along the length of the sample. An example of the principle of FSM is shown in Figure 22.

The ‘signature’ relates to the first measurement that determines the base-line potential difference pattern that all other measurements are compared to in order to estimate the change experienced by the sample [89]. This unique, ‘signature’, potential field is set up in the sample based on the geometry and several other parameters.

Metal loss or other structural damage will cause an increase in electrical resistance at that point due to a decrease in thickness. Hence the potential difference measured between two points will change.

Advantages of this technique include the ability to monitor large structures with an ability to control resolution; increased resolution can be achieved by increased number of pins per meter. Furthermore, it is a technique that can distinguish between localised and general corrosion and allows detection of corrosion in real time, often quicker than other methods [87]. It has also been shown to be effective at detecting cracks forming in bridge components [88].

Unfortunately, as this technique relies on electrical measurements it would only be applicable to the underlying substrate as the coating is non-conductive. Connection to the substrate during measurement is also required which, therefore, would require penetration of the coating layer. This would likely have a negative impact on the coating performance.

## 10. Electrochemical Techniques

### 10.1. Potentiodynamic Polarisation Techniques

**Figure 23:**
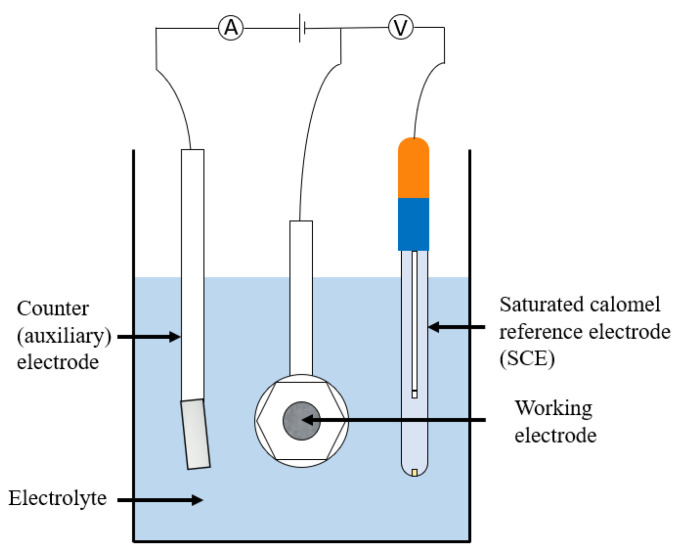
An example of a potentiodynamic experiment set up.

This title covers a number of techniques which, although are mainly used in lab-based testing, can occasionally be applied to in situ measurements. They allow determination of highly important corrosion parameters and are often used for analysing passivating metals. In potentiodynamic polarisation three electrodes are used. The voltage of interest is measured between a working and reference (often a saturated calomel) electrode whereas the current of interest is measured between the working electrode and a counter (often platinum) electrode. A standard set up is shown in Figure 23 and a list of tests that can be carried out is given in Table 6.

### 10.2. Harmonic Analysis

A limitation of the polarisation methods, and other methods that make use the Stern–Geary equation, is that it is dependent on the accuracy of the Stern–Geary coefficient, B. This introduces some inherent uncertainty in any corrosion rate calculation as this coefficient is not a constant but is often taken to be one [98,99].

Several methods described by [98] exist that can reduce this uncertainty, but the harmonic analysis technique is unique in that it directly measures, for the system being investigated, the Stern–Geary coefficient [98].

In harmonic analysis a sine wave is applied to a corroding electrode and the distortion due to corrosion results in production of harmonics of this sine wave; the Tafel constants and hence the Stern–Geary coefficient can be determined by analysis of the amplitudes of the harmonics produced [98,99].

The Stern–Geary equation with derived coefficient from Tafel lines [100] is given by:

(4) $$i_{corr}=\frac{\beta_{a}\beta_{c}}{2.303\left(\beta_{a}+\beta_{c}\right)}\frac{1}{R_{p}}$$

where *β_a_* is the anodic Tafel slope and *β_c_* is the cathodic Tafel slope. Hence, the advantages of this method are that it does not presume values for the Tafel constants and it is also reported that measurements can occur quicker than other electrical techniques [100].

The disadvantages are that more complex methods of stimulating and measuring the test sample are required and hence more equipment and control systems are required. It is also the case that data analysis is more complicated.

### 10.3. Electrical Impedance Spectroscopy (EIS)

EIS is an AC technique and uses small potential excitation amplitudes to cause minimal perturbation of the sample [96]. It can provide information on both electrode capacitance and resistance and allows more easy resolution of variables than DC techniques. It involves the measurement of impedance between corroding metal and a reference across a wide frequency range [77] and is ‘one of the main methods to evaluate performance of coatings’ [101]. Impedance is the Alternating Current (AC) equivalent of resistance. Hence, can be stated in a way similar to Ohm’s Law [96]:

(5) Ê=ÎZ

where Ê is the amplitude of potential sine wave, Î the amplitude of current sine wave and *Z* is the impedance (Ωhms)

**Figure 24:**
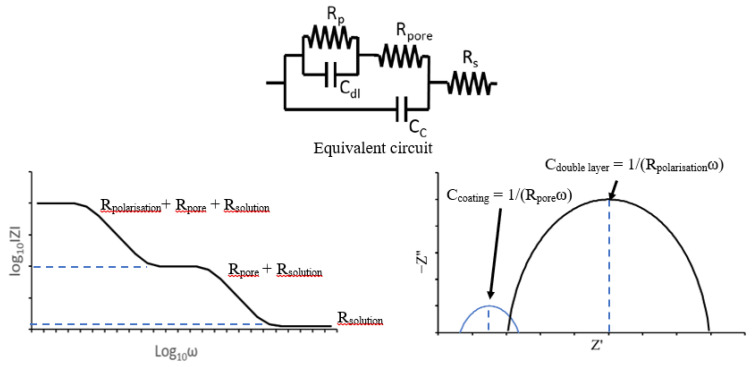
Example Bode (left) and Nyquist (right) plots for a coated metal electrode under EIS testing modelled on the equivalent circuit for a coated metal electrode (top).

Often EIS is used to model an electrochemical cell as an electronic circuit in order to use established circuit theory to analyse it [102]. Examples of physical electrochemical properties able to be represented in EIS are electrolyte resistance, polarisation resistance, coating capacitance and double layer capacitance. Through analysis of the plots gained by EIS experimentation these properties can all be calculated for the test piece. An understanding of the performance of the system can then be obtained. There are two main plots used in EIS analysis, Nyquist and Bode [96] and examples of these plots from analysis of a coated metal is shown in Figure 24.

Nyquist plots show imaginary impedance component (−Z”) against the real impedance component (Z) whereas Bode plots the log of impedance magnitude (log(Z)) against log of the frequency (log(ω)) where [96]:

(6) ω=2πf

where f is frequency in Hz. EIS uses three electrodes; working, counter and reference, and the polarisation applied to the working electrode is a small amplitude sine wave. By varying the frequency of the sine wave and measuring the current flowing, the impedance of the electrode as a function of frequency can be determined. This is the electrochemical impedance spectrum.

Calculation of the polarisation resistance can allow calculation of the corrosion rate, via the Stern–Geary equation, in a similar way to that in linear polarisation resistance. However, other properties of significant interest can be calculated such as the coating capacitance; this exists when two conducting materials are separated by a non-conducting material and is defined as [102]:

(7) $$C=\frac{\varepsilon_{o}\varepsilon_{r}A}{d}$$

where C is capacitance, ε_o_ is permittivity of free space, ε_r_ is a dielectric constant, A is surface area of one plate, d is distance between plates. Coating capacitance is a useful parameter for establishing the condition of a coating and monitoring degradation as it allows measurement of the thickness of the organic coating. Furthermore, the change of a substrate over time can be measured such as the absorption of water through a change in the capacitance due to a change in ε_r_. Pore resistance is another variable that can be calculated by the EIS method; this is able to give information about the integrity of organic coatings.

Hence the main advantage of EIS is the quality and amount of information that can be gathered as to the condition of the coating and substrate in a relatively quick manor. It has also been the subject of extensive research and therefore is well developed and optimised for several different situations. For example, EIS can be used to analyse blister formation, permeability to water, coating swelling, delamination and corrosion [102,103].

The disadvantages of EIS are that it requires three electrodes and immersion in and electrolyte [101]. Although there have been efforts to produce in situ, electrolyte free kits [104], complexity in design still exists. Furthermore, the majority of EIS calculations use the non-linear least squares fitting algorithm to fit the data to the expected spectrum. This has some limitations caused by incorrect modelling poor data fittings, poor initial values and noise.

There has been development of specific sensors for diagnosis of polyurethane organic coatings [101]. A miniature battery powered sensor example is given in [101] and this was shown to be a sensitive and effective method of determining coating performance on an in situ basis [101].

The authors of [105] have proposed a design for an EIS which functions in atmospheric conditions negating the immersion requirement. This has been shown to give consistent data when measuring a coated sample [105]. Other designs have also been suggested that allow long range, low cost remote monitoring [106,107].

It has also been shown that EIS can use two embedded sensors as the working and reference electrodes. This has the benefit of not requiring electrical contact to the substrate and hence does not fully penetrate the protection, offered by the coating, with wires [108]. This also allows different parts of a coating system to be analysed separately [109].

### 10.4. Electrochemical Noise

Electrochemical noise refers to the fluctuations in potential and current that occur during corrosion of an electrode [77,98]. Theoretically, by studying the ‘noise’ created by a corroding element, information can be gathered as to the corrosion process itself [98]. For monitoring applications results are analysed statistically. The authors of [110] concluded that this technique could ‘offer much promise for the detection of corrosion’.

During testing it is possible to measure both potential noise and current noise. EPN (electrochemical potential noise) is either measured between one electrode and a reference or two electrodes. ECN (electrochemical current noise) is measured between two electrodes or by holding a fixed potential of a single electrode [98]. Alternatively, it is possible to simultaneously measure both with a three electrode setup [98].

Directly related to the fluctuation amplitude is the standard deviation; this is the most commonly used statistical analysis technique. Eden suggested the following relationship between standard deviation and a quantity electrochemical noise resistance [98,111]:

(8) $$R_{n}=\frac{\sigma_{E}A}{\sigma_{I}}$$

where *R_n_* is the electrochemical noise resistance, *A* is the area of the sample, *σ_E_* is the standard deviation associated with the potential and *σ_I_* is the standard deviation associated with current [98]. Further work has shown that the linear polarisation resistance, *R_p_*, is comparable to the value of *R_n_* [98]. Hence, using the Stern–Geary equation, icorr can be calculated (assuming *R_p_* is approximately equal to *R_n_*):

(9) $$i_{corr}=\frac{B}{R_{p}}\;=\frac{B}{R_{n}}\;$$

where *B* is the Stern–Geary coefficient and *i_corr_* is the free corrosion current [98]. It is possible to estimate the tendency for localised corrosion to occur, using a method which estimates the true coefficient of variation, which was developed by Kane [98,112]

(10) $$TCR=\frac{\sigma_{I}}{I_{corr}}$$

where *TCR* is termed for the true coefficient of variation. Using several assumptions, Schottky demonstrated theoretically that [98,113]

(11) $$\sigma_{I}=\sqrt{2qIb}$$

where *q* is the charge on an electron, *I* is the average current and *b* is the bandwidth of the material [98]. Hence it can be shown that:

(12) $$q=\frac{\sigma_{I}\sigma_{E}}{Bb}$$

The advantages of EN include the ability to determine the type of corrosion occurring, although this is dependent on certain conditions [98]. Furthermore, noise measurements have been shown to be well correlated with EIS when determining corrosion resistance of organic coatings [114]. The technique is a non-interfering method and hence does not affect the corrosion process unlike some DC techniques [114]. Unlike EIS, EN can give an indication as to the localisation or uniformity of the corrosion which is often useful [114]. Finally, Woodcock et al. (2004) developed a no connection to substrate (NOCS) method leading to easier measurement of coatings [114].

The disadvantages of this technique are that it requires a high sensitivity measuring system or amplification of signals. Both of these solutions have errors associated with them [98]. Additionally, it is difficult to conclude with confidence how corrosion is occurring, even though the rate of corrosion can been measured with reasonable accuracy [98]. Often, other techniques involving measurement of *R_p_* are more reliable and produces less noise and this technique is also very sensitive to interference [98].

Several reviews into EN have been carried out and it has been concluded that EN is a versatile, quick and simple technique for determining the defects present in coatings and the level of protection afforded [115,116]. However, it is suggested that in systems with passivity and inhibition the data analysis is more difficult and that data collection and analysis methods are crucial to the effectiveness of the technique [115,116].

Mills [117] examined Alkyd and Polyurethane coatings using the NOCS technique. It was suggested that areas may be able to be resolved in terms of their resistances allowing spatially resolved data points. By using copper pads the requirement to attach other reference electrodes such as a SCE (saturated caramel electrode) was avoided [117].

### 10.5. Scanning Electrochemical Microscopy (SECM)

**Figure 25:**
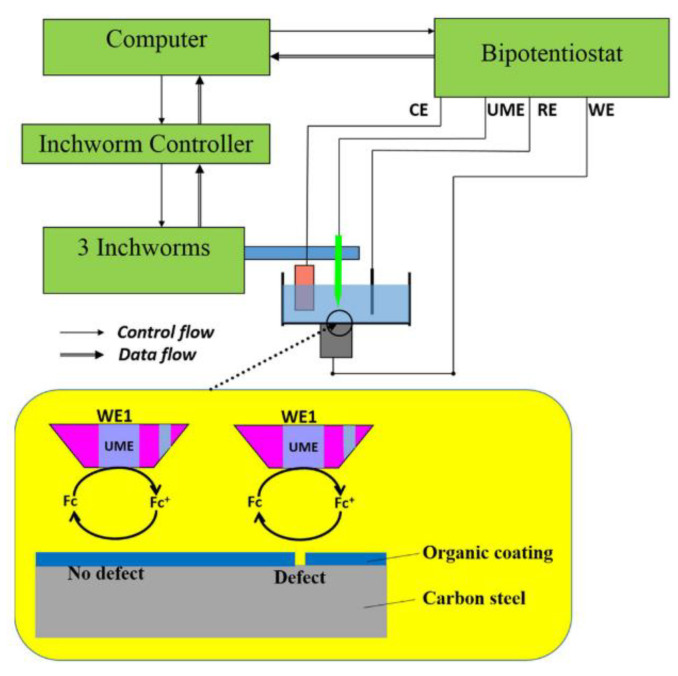
Example SECM (Reprinted with permission from ref. [121]. Copyright 2019 Elsevier).

This is a technique that has been described to ‘provide spatially resolved (electro)chemical information about a sample under investigation’ [118]. The working principles and methods are well described by Wittstock [119]. It relies on measuring, at the surface of a sample, the rate of redox reactions in order to determine corrosion properties [118]. Unlike SVET (the scanning vibrating electrode technique) it can determine the nature of chemical species involved in the process and there are examples of it being used for coated metallic products documented in [120]. The cathodic reaction can be measured in situ within the coating defect [120] and it allows the determination of the reactivity of a point giving an indication of the degradation present at that position [121]. The experimental set up is shown in basic form in Figure 25.

Whilst this technology has the potential to provide a large quantity of useful data at high accuracy, it is difficult to consider how in situ measurements may be made outside of a laboratory. Furthermore, automation and the likelihood of disturbance from other factors leads to the conclusion that this technique may not be useful for the specific application at the heart of this work.

## 11. Other Techniques

### 11.1. Embedded Strain Gauges

These are commonly used to measure force, or strain, and are sensors that can be made from a variety of substrates [122]. They allow measurement of strain through a corresponding change in their electrical resistance, known as piezoresistivity. Hence, measurement of deformation or movement can be determined [122].

It can be shown that:

(13) $$\frac{\Delta R}{R_{0}}=GF\varepsilon$$

where Δ*R* = *R* − *R*_0_ where *R* is the resistance at strain *ε* and *R*_0_ is the resistance at strain zero (initial conditions). *GF* is the gauge factor which defines the sensitivity of a change in resistance with strain [122,123]. The strain in the device can be calculated by:

(14) $$\varepsilon \left(x\right)=\frac{3hx\delta }{2l^{3}}$$

where *l* is length, w is width, *h* is thickness, *δ* is vertical displacement, longitudinal strain is *ε* at position *x* [122].

**Figure 26:**
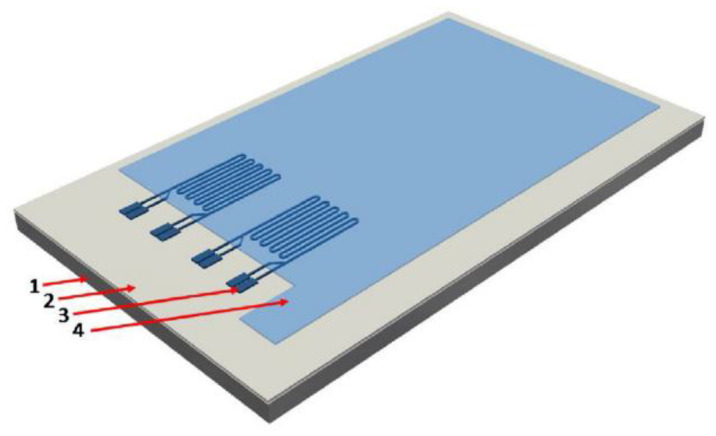
Example of a screen-printed strain gauge embedded in an organic coating. (Reprinted with permission from ref. [123]. Copyright 2018 Elsevier).

Strain gauges have the advantage that they can be screen printed, as described by [122], and hence they could be placed on any layer of the coating such as the topcoat or primer [122]. This gives a potentially easy route to deployment at a large scale. Design and printing considerations are given in [84] in which it was concluded that organically coated steel ‘can be used as a substrate for printed electronics’. Screen and inkjet printing were both considered by [84] but it was reported that screen printing was more appropriate for thicker conductors, due to larger depositions. An example of a printed strain gauge under an organic coating is shown in Figure 26.

The conductive inks used [123] in the studies carried out were a Loctite EDAG PR 406B E&C (a carbon black thermoset resin) and an EDAG PF 050 E&C (a silver thermoplastic). These have cure times of 30 min at 150 °C and 3 min at 140 °C and flash points of 54 and 74 °C, respectively [124,125]. However, even when the top coat was applied to these strain gauges and cured at 250 °C for 90 s, there was no suggestion that this excessive temperature affected the strain gauge operation [123].

A full report by Enser in 2018 [126] describes the feasibility of integrating capacitive, strain and piezo and pyro electric sensors into organic coatings on steel products. Perhaps optimistically, this reports that ‘the concept has the potential to introduce additional functionality [[] without the need of significant changes to associated production processes’ [126]. It is possible that one or more the of the sensors described in this report may provide significant coating integrity information.

There is also significant current development of conductive inks; one notable ink is a conductive epoxy which contains silver flake pigment. One manufacturer (XYMOX) states that although having a cure condition of 130 °C for 10 min, the flash point is much higher at 212 °C suggesting that the survivability of the electronics may be increased [127].

The advantages of this technology are that it is relatively simple, however, when embedded in a coat, can provide a good indication of the coating condition. Unfortunately, it is unknown if embedding the sensor reduces the protective properties of the coating and it is difficult to consider how to make an electrical connection to the sensor.

### 11.2. MEMs Based Systems

A MEMs is a micro-electromechanical system; these are tiny devices that can be used as a sensor or control factors on a scale much larger than their own. They are composed of a mixture of electrical and mechanical components [128] and they use mechanical motion to influence an electrical signal.

The basic design is a silicon chip composed of microsensors and microelectronics and these sensors can be configured to measure chemical, electromagnetic or mechanical changes [128]. They have a high diversity of potential uses due to their small size and volume and some examples of in-use application include accelerometers, data storage and air bag deployment [128].

Domains that can be measured include:

- Mechanical—force, pressure, velocity, acceleration, position;
- Thermal—temperature, entropy, heat, heat flow;
- Chemical—concentration, composition, reaction rate;
- Radiant—electromagnetic wave intensity, phase, wavelength, polarisation reflectance, refractive index, transmittance;
- Magnetic—field intensity, flux density, magnetic moment, permeability;
- Electrical—voltage, current, charge, resistance, capacitance, polarisation.

The advantages of using these sensors is that they have high sensitivity and a long life. They are also rugged, small and are relatively inexpensive [129]. Additionally, many are commercially available and can be bought tailor made to measure a number of features to a stated degree of accuracy. Some MEMs have been developed which measure the diffusion rate of a corrosive species through a sensor and as the sensor corrodes its resistivity changes and this can be detected [129]. It is assumed a sensor based on measuring several factors could be developed and it may be the case that a previously mentioned technology could be facilitated through the use of MEMs.

The disadvantage of these systems is that they are often complex to design, and communication and powering of the device may be complicated in situ. In order to detect certain features, the device may have to have an electrical connection to the substrate which would require penetration of the coating.

### 11.3. Nuclear Thin Layer Activation

For this technique a small section of material is exposed to a high energy beam of charged particles, to produce a radioactive surface layer. The loss of material, as a result of corrosion of this layer, is measured by considering the corresponding decrease in radiation intensity [130,131]. Radiation levels are low enough for safe handling with only basic precautions, although this technique is often viewed with concern [131].

The direct relationship between thickness reduction and measured radioactivity allows calculation of the thickness, however, complicated calibration is required and it is a so called ‘non-direct method’ [130]. An example of this principle in use is a study where a gamma radiation detector was used to monitor changes in a carbon steel irradiated with a high energy proton beam. The isotope formed was Cobalt 56 [130].

Carbon and stainless steels have also been tested with this method and the results are stated to have correlated with gravimetric testing [131]. However, some samples’ radioactivity change was below the sensitivity threshold, leading to a lack of measurement. The conclusion of this work was that this is a method that is viable for the measurement of material loss and measurement of specific locations to give insight into the areas most affected [131].

If irradiating larger areas of samples, it is of crucial importance to ensure that the level of activation is uniform throughout the sample in order to have repeatable readings [132]. A scanning beam may achieve this and some work has been done using TLA on a macroscopic level; the advantage of this technique is the ability to use it on curved surfaces [132]. The measurement can also be carried out without damaging the coating in any way.

Consideration of the half-life is important, if long term measurement is required, as is the penetrating power of the radiation, should the sample be imbedded in such coating [132].

### 11.4. RFID Technology

**Figure 27:**
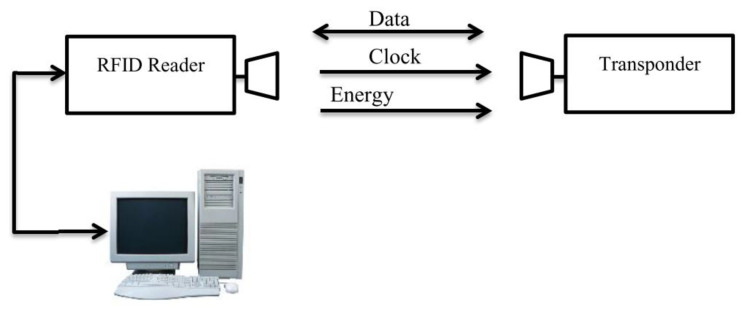
A basic description of the RFID system. (Reprinted from ref. [135]).

Typically, RFID technology is composed of an antenna, a reader and a tag [133]. The tag or chip contains the memory, energy harvesting unit, microcontroller, and response generator [133]. The components of an RFID system function as a coupling device, an interrogator and a transponder, respectively [134]. The tag receives an electromagnetic wave from the interrogator via the antenna. Data in the form of, for example, an identifier code can then be supplied by the tag via the electromagnetic wave [134]. This process is shown in Figure 27.

RFID is similar to a barcode technology, in terms of the data transferred, but does not require line of sight. The reader has a microcontroller, signal generator and a signal receiver/detector and an antenna for each. Passive tag systems have an antenna, a transponder, rectifier circuit, controller and memory. LF (Low Frequency), HF (High Frequency) and UHF (Ultra High Frequency) frequencies can all be used for the operation of the tag depending on various requirements. The higher the frequency the greater the distance of detection and depending on the frequency the type of coupling that is employed changes.

LF and HF works on inductive coupling (near field coupling) where the reader powers the tag, synchronisation of clock and a carrier for return data. The field emitted by the reader couples with the tag and a voltage is induced; this is used as power for memory access. When a load is connected the impedance can be used to change current and hence the rate of change of current can be used to create a voltage. The load can be controlled and switched on and off to create binary communication and hence changes voltage across tag coil affecting the carrier wave of the reader. High frequency can be read from approximately 0.8 m but is generally a cheaper solution [135].

UHF uses far field coupling and has a range of up to several meters. The reader sends a continuous signal to tag and the tag sends weak signal (backscattered signal) back. Depending on the load matching across coil, the intensity of backscattered signal changes. By changing load condition, the intensity of backscattered signal can be controlled. This is known as a backscattered modulation/coupling technique and in order for this to be effective the initial signal from reader is required to be strong. UHF can be read from approximately 15 m away but is generally higher cost [135].

**Figure 28:**
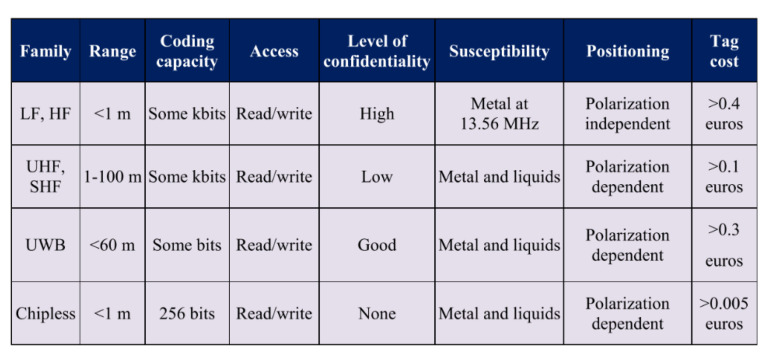
Classification of RFID technologies by frequency (Reprinted with permission from ref. [133]. Copyright 2016 Elsevier).

These different systems are shown in Figure 28.

Three types of tag also exist:

A passive tag has no internal power and is solely powered by harvesting the reader’s electromagnetic field [133]. With an effective range of approximately 20 feet they are often cheaper than active tags and hence are often disposed with the product [134]. Passive tags vary the load impedance connected to the antenna. This affects the amplitude of the signal reflected back to the reader allowing communication through load modulation [136]. A semi passive tag uses harvested energy to respond to the reader [133]. A reader is used to wake a battery powered tag and hence there is extended battery life over an active tag [134]. An active tag has its own power source in the form of a battery and uses this to emit radio waves to a reader [133]. This allows larger range and a better degree of accuracy in detection. However, there is the obvious disadvantages that the tags are generally larger and more expensive and also have a finite lifetime [134].

There are three types of data storage:

RO or read-only is a system where there is stored data that cannot be changed or removed [134]. RW or read-write is a system where the stored data can be edited or removed [134]. WORM or write once, read many is a system where the data can be changed or removed once only [134].

Various corrosion based radio frequency devices have been developed, often for measuring the diffusion of water through concrete, and they work on the principle that the sensors electromagnetic performance will vary as a result of the quantity of water surrounding it [137,138]. This process is based on the principle that the permittivity of a medium can be determined through analysis of the impedance of an antenna that is embedded in that medium. Capdevila [137] describes the methodology behind this technique in detail.

Use of RFID technology to determine the water diffusion through a coating has also been considered. Khalifeh et al. (2016) suggested that to overcome the sensitivity of a thin layer coating a new approach is required, such as the use of ‘microstrip planar resonators’ which have a smaller field of electromagnetic influence. It was shown that an RFID tag can be embedded in the coating and that, with background testing, chemical and physical changes can be detected [138]. It was concluded that this is ‘a promising and reliable tool for the monitoring of protective properties of coatings’ [138].

The benefit of these is that they are passive devices and hence complex issues involved with powering the device are negated. It is also reported that a standard RFID reader can be used with these sensors reducing the technical demand [137].

The disadvantage of RFID based systems are that they are heavily affected by metal interference and require complex design to ensure optimum range. Furthermore, complexities exist in combining the communication system into a sensing system.

### 11.5. NFC Technology

Near field communication (NFC) is an RFID compatible interface and protocol which was developed with the aims of simplifying the RFID system [139]. This allows communication wirelessly between devices at a distance of approximately 10 cm or less, making it one of the shortest range wireless communication protocols [135]. It uses RFID technology and hence communication is based on the principle of magnetic coupling and can involve passive or active tags.

The main advantages of NFC, over RFID, are that reading devices are relatively simple and are now commonplace in mobile phones unlike RFID readers which are more complex. Data sharing is also relatively simple via the protocol [135].

An NFC device is classed as active if, similar to the RFID system, it is capable of generating a radio frequency field. If this is not the case it is classed as passive and hence transfers data via the inductive coupling method [135]. Communication is active if achieved by two active devices and passive if one is passive and one active. Instead of being called the reader and tag, as in RFID, the devices are known as the initiator and target.

### 11.6. Biological Detectors

The aim of these sensors is to detect the formation of biological films on the surface of coatings. As previously mentioned, these are detrimental to coating life.

There has been some success in detecting biofilms by using a hydrogen peroxide-based detector which reacts with biofilms if present to form bubbles. There has also been some success in using a colouring in this peroxide in order to increase the ease of detection [140].

### 11.7. pH Sensors

A number of options exist for detecting changes in pH. There are, for example, standard electrodes which could be used for comparison, such as glass electrodes, yttria stabilised zirconia, metal oxides. These are all explained in detail in [141]. However, while commonplace in laboratory testing, their suitability for in-field coating monitoring is very limited.

Some corrosion specific sensors have emerged from research. For example, Behnood et al. (2016) discuss an embedded potentiometric electrode and fibre optic sensors for monitoring alkalinity in concrete structures [142]. To overcome the issue of electrodes involving glass, there have been a number of developments such as solid state, hydrogel film and fibre optical sensors. Metal–metal oxide sensors which calculate pH based on the redox reaction reversibility show some promise as in situ sensors and a variety of fibre optic techniques exist as well; these use a variety of factors such as refractive index, absorbance or fluorescence to determine pH values. These methods are described further in [142].

Potentiometric thick film sensors are presented in [143]. Similar to the chemical sensors explained below, these are screen printed sensors for measuring pH in which a sliver–silver oxide (Ag/Ag_2_O) sensor is used. Good response time and repeatability was seen with these sensors which were developed for monitoring concrete environments [143].

While pH monitoring could be a suitable method for monitoring corrosion the difficulties involved in using this, usually aqueous reference comparison technique, make it less suited to this specific application.

### 11.8. Chemical Sensors

**Figure 29:**
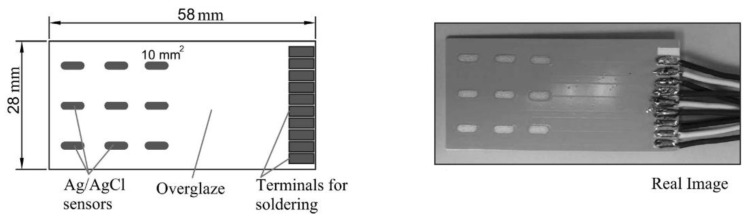
Design of a potentiometric chlorine sensor. ((Reprinted with permission from ref. [144]. Copyright 2016 Elsevier).

A potentiometric chlorine thick film sensor for concrete monitoring is outlined in [144]. These are small sensors with a number of silver/silver chloride sensors attached, as shown in Figure 29. Ag/AgCl is used as it can be used to detect changes in variations in Cl^−^ concentration [144]. It was shown these sensors could be screen printed and provide reasonable accuracy at low cost and ease of manufacturing [144].

A wireless chlorine sensor for monitoring of concrete structures is described by [145]. This involves using a silver/silver chloride reference electrode with a wireless interface. As the concentration of chlorine ions changes, the half-cell potential of the electrode changes, and this is measured by the circuitry.

The main disadvantages of these techniques are that they currently require a reference and working electrode set up in an electrolyte. Whilst in-lab results, and monitoring, are therefore possible, and ‘accurate’, it is difficult to reproduce these results in situ and the equipment complexity increases dramatically. Additionally, they only provide an understanding of the environmental conditions rather than the actual current performance of the coating.

A range of other chemicals are able to be detected using various methods which are summarised in [146] although not explained in any detail here.

## 12. Analysis of Techniques

Table 7 appraises each technique based on several criteria. The applicability column aims to state how suitable the technique is to this application as well as whether the technique has been used currently for the monitoring of coatings, whether that be in the lab or as a deployed system.

## 13. Conclusions

There is no doubt that corrosion is a big problem for the construction industry. However, whilst the industry understands the benefits of protecting metal components against corrosion, there appears to be little in situ monitoring of the most common protective method; organic coatings. Reinforced concrete has been shown to be the current main focus of in situ testing development and it seems that for many companies a solution involving a human operated probe, such as regular ultrasonic testing, is currently sufficient. Despite this, there is a clear appetite for smart solutions that allow monitoring of larger, building scale, organically coated components. Embedded sensors in paint systems are being considered, however, there is a realisation that embedment of systems may lead to a decreased coating performance. A serious complication is that many techniques, that are effective in the lab, require either complex and delicate reference electrode systems or electrical connection to both the substrate and surface, producing a potential coating breach. Furthermore, difficulties exist in powering and collecting data from a device without significantly penetrating the barrier produced by the coating. It is perhaps because of these features, passive sensors such as corrosion indicating paints and RFID style sensors are emerging as a promising new area of research.

Unfortunately, there seems to exist an inevitable dilemma in current research in which a technology fits into one of the following categories:

Simple and non-intrusive to the coating. In this case the data received is equally basic in nature and is often only reactive to failure offering little or none of the expected benefits of in situ monitoring. An example is corrosion indicating paint.

Complex and expensive. The sensor requires a high-resolution or other expensive component or is complex to automate. An example would be infrared thermography.

Simple and low cost. Although easy to implement and use, the sensor does not provide sufficient quality or depth of data to allow confident analysis. An example would be corrosion coupons.

Complex and intrusive to the coating. Whilst the data produced is very useful, the coating integrity is affected by the sensor and hence overall the sensor decreases the effectiveness of the coating. Examples of these technologies include capacitance and EIS sensors.

Therefore, a clear motive exists to develop or refine a technique that produces the quality of data of a complex, expensive or intrusive technique but does not suffer from the limitations currently posed. While modification of techniques used frequently in the oil and gas industry may provide the easiest route to a solution the complexity of automating sampling is vastly greater in this specific application. Therefore, it is expected that the optimum solution will require novel modification of an existing solution or development of a new solution.

## Figures and Tables

**Table 1:** Coating components.

Component	Function	Influences	Examples	References
Resin	Is the backbone or structure of the coating and forms the continuous film	Mechanical properties, flexibility, adhesion	Organic polymers such as polyester and polyurethane	[9,11,12]
Solvents	Acts as a medium for the resin to be dissolved in for application. Allows even dispersion of particles	Fluidity, viscosity, coating method, adhesion, durability	Acetone, IPA	[9,10,11,12]
Pigments	Provide colour and/or functionality	Colour, UV and corrosion protection	Zinc dust, phosphates	[9,10,11,12]
Fillers and additives	Increase coating weight and decrease cost. Ensure coating properties	Cost, foaming, wettability, stability	Talc, lime, surfactants	[9,10,11]

**Table 2:** Coating layers.

Layer	Thickness	Properties and Function	References
Pre-treatment	=<1 µm	Acts as a corrosion barrier, inhibitor and adhesion promotor. Allows mechanical keying in of subsequent layers	
Primer	≈5–15 µm	Promote adhesion, provide a homogeneous layer for subsequent layers and reduce the effects of metallic surface roughness. Increases surface energy, wetting and removes impurities. Often contains corrosion inhibiting pigments	[14,15]
Topcoat	≈15–200+ µm	Provides the final colour and gloss via pigment selection. Protects against chemical and UV degradation and water ingress to other layers	[13,14]
Clear coat	≈15–50 µm	Used where high gloss or additional protection is required. Usually transparent, it is also used to improve mechanical properties such as scratch or abrasion resistance	[16,17]

**Table 3:** Factors contributing to coating degradation.

Family	Factor	Brief Description	References
Environmental	Light	High energy photons in the UV spectrum degrade organic coatings through methods such as photooxidation and bond scission causing fading and embrittlement. Photons produce oxidising holes in semi-conductive pigments which mineralise organic coatings producing pores and chalking.	[8,19]
	Temperature	High temperatures can soften thermoplastic coatings, cause cracking, rupture bonds and increase permeability. Cold temperatures introduce brittleness. Fluctuations can cause ruptures, cracking or delamination due to differences in substrate and coating expansion rate.	[8,19]
Mechanical	Internal stresses	Often formed during curing shrinkage or due to coating thickness variations, these increase the likelihood defect formation.	[19]
	Abrasion	Brittle paint systems are susceptible to coating removal via abrasion from wind driven particles such as dirt or sand.	[19]
	Erosion	General degradation of a coating through wear over time that is accelerated via adverse weather.	[18,20]
	Impact	Accidental damage often caused during installation or when moving parts are present. Any breach of the coating exposes the substrate to corrosion.	[19]
Chemical	Water	Water can penetrate pores or cracks and separate bonds, cause expansion of the coating and hydrolyse functional groups. Penetration can be accelerated through electroendosmosis or osmotic pressure.	[19]
	Solvents	If not fully removed during the curing process these can soften coatings and increase water penetrability.	[19]
	Acids and alkalis	Acids attack susceptible bonds such as urethane and promote corrosion rate. Alkalis can degrade ester linkages through saponification.	[19]
	Gasses	Oxygen contributes to a variety of free radical degradation reactions which can lead to depolymerisation and chain fragmentation.	[8,19]
Biological	General	Biological matter can hold or produce moisture and a variety of chemicals close to the surface which cause accelerated degradation.	[8,19]
	Mildew	Oil-based and coatings containing fatty acids can undergo direct attack. Components of coating systems can be directly consumed or degraded by enzymes.	[19]
	Marine fouling	When microorganisms attach to submerged samples, they can cause fluid flow abrasion and can contribute to the formation of a differential aeration cell.	[19]

**Table 4:** Under film corrosion phenomenon.

Family	Method	Brief Description	References
Blistering	General	‘Local separation of the coating from the substrate’, blistering occurs when adhesion strength is low and can rupture. Internal blister conditions are often corrosively aggressive.	[8,20]
	Osmotic	Substrate salt or oil contamination produces the driving force for osmosis to draw in water through the permeable coating.	[8,20]
	Anodic	Corrosion occurs at a defect site and the production of iron oxide from ion produces the blister. Cathodic activity occurs at the blister edge leading to cathodic disbondment.	[19]
	Cathodic	Corrosion occurs at a defect site and cathodic activity shifts under an oxygen permeable coating. OH^−^ groups and electro-endosmosis draw water into the cathodic site which lifts the coating.	[19]
	Thermal	Condensation occurs if the substrate is cool compared to external humid air. Formation is usually aided by a pre-existing defect.	[20]
Anodic	Filiform	Distinguished as small tendril like patterns often radiating out from a defect such as a scratch. Formed by an active, acidic head, which proceeds under a coating, and an aerated alkaline tail.	[8,10]
	Undermining	Anode creeps under a coating from a defect or edge leading to a differential aeration cell. Cl^−^ migration can lead to decreased pH values and aggressive attack at the anodic site.	[10,20,21]
	Wedging	Production of corrosion product mechanically forces the coating away exposing fresh metal. Present with poor adhesion.	[10,19,22]
Cathodic	Disbondment	When the cathodic reaction is concentrated under the coating the OH^−^ species decrease adhesion, form alkaline compounds, attract further water ingress and promote electroendosmosis.	[10]

**Table 5:** Current techniques for corrosion and or coating monitoring.

Family	Family/Technique	Technique
Non-destructive	Wave based	Acoustic emission
		Ultrasonic
		Terahertz waves
	Magnetic	Magnetic flux leakage
		Magnetic adaptive testing
		Magnetic memory method
	Thermal	Pulsed thermal NDT
		Infrared thermography
		Eddy current pulsed thermography
Optical	Visual	Corrosion indicating paint
	Fibre based	Stressed optical fibres
		Braggs gratings
		Corrosion product detection
		Interferometry
Mass		Corrosion coupons
Electrical		Galvanic
	Resistance	Zero resistance ammetry
		Electrical resistance
		Induction resistance probes
		Capacitive sensor
		Electrical field signature method
Electrochemical		Potentiodynamic polarisation
		Harmonic analysis
		Electrical impedance spectroscopy
		Electrochemical noise
		Scanning electrochemical microscopy
Other		Embedded strain gauges
		MEMs based sensors
		Nuclear thin layer activation
	RF based	RFID technology
		NFC technology
	Conditions	Biological detectors
		pH sensors
		Chemical sensors

**Table 6:** Potentiodynamic polarisation tests.

Test Type	Brief Explanation	Used for	Procedure	References
Anodic polarisation	Sample is forced to become anode	Determining pitting, galvanic and localised corrosion behaviour in active-passive metals	ASTM G5	[90,91]
Cathodic polarisation	Sample is forced to become cathode	Likely rates of corrosion and kinetics of the system	ASTM G59	[92]
Cyclic polarisation	Sample potential is increases until the current is 5 mA then reversed	Tendency of putting and determining breakdown, passivation and protection potentials	ASTM G61	[93]
Cyclic voltammetry	As above but a set voltage is scanned to rather than current	Tendency of putting and determining breakdown, passivation and protection potentials		[94]
(Cyclic) Galvano-staircase	Current is increased then decreased in steps	Determining protection potential. Gives qualitative, quick sensitive data	ASTM G100	[93]
Electrochemical reactivation	Potential is varied and current measured	In situ corrosion, sensitisation of stainless steels		[95]
Potentiostatic polarisation	A single potential is applied, and current measured	Resistance to pitting	ASTM G150ASTM F746	[93]
Tafel extrapolation	Analysis of potentiodynamic tests	Corrosion potential, corrosion current density	ASTM G5	[93]
Linear polarisation	Current is measured as voltage is varied linearly	Free corrosion current, polarisation resistance	ASTM G217	[69,71,77]
Galvanostatic pulse	Pulsed current is applied galvanostatically and potential is measured	Polarisation resistance, free corrosion current polarisation potential		[96,97]

**Table 7:** Technique appraisal.

Technique	Detectable	Not Detectable	Pros	Cons	Powered	Automation	Ease of Installation	Applicability
Acoustic emission	Initiation and propagation of flaws	Any defect that does not lead to emission of sound waves	Cumulation assessment of damage possible. Locational pinpointing of damage. Determine active flaws	Very sensitive to outside factors. Easily affected by noise. Difficulty in sizing defect or determining severity	Yes	Yes	Med-Low	Currently a deployed system for monitoring corrosion in other applications. Promising lab testing for this application has been carried out
Ultrasonic	Thickness measurements, delamination, cracks, blisters and corrosion product	Changes in colour	Well developed technology. Affordable and reliable. Large range of flaws detectable	Affected by surface cleanliness and noise	Yes	No	Med-Low	Currently a deployed system for monitoring coatings in other applications. Promising lab testing for this application has been carried out
Terahertz waves	Thickness, changes in refractive index and absorption coefficient	Changes in colour	Better for defect prone coatings that are thicker. More suitable for opaque coatings	Affected by surface cleanliness and noise	Yes	No	Med-Low	Currently a deployed system for monitoring coatings in other applications. No investigation for this application
Magnetic flux leakage	Thickness, cracks, flaws	Non-magnetic layers	Reliable and accurate, can measure through an organic coating. Large range of flaws detectable	Affected by surface cleanliness and noise. Unknown if coating flaws or degradation can be detected	Yes	No	Med-Low	Currently a deployed system for monitoring corrosion in other applications. Promising lab testing for this application has been carried out
Magnetic adaptive testing	Flaws in substrate	Non-magnetic layers	Types of flaws can be determined	Unknown if coating flaws or degradation can be detected. Requires lengthy calibration. Newly developed technology	Yes	Yes	Med-Low	Currently an early-stage technique for monitoring corrosion in other applications. No investigation for this application
Magnetic memory method	Structural damage and flaws	Non-magnetic layers	Can use ambient magnetic fields. Can determine locations of interest for further testing	Does not produce quantitative data. Newly developed technology. Not as accurate as other similar methods and prone to noise	Yes	Yes	Med-Low	Currently an early-stage technique for monitoring corrosion in other applications. No investigation for this application
Eddy current testing	Thickness measurements, flaws and defects	Non-conductive layers	Sensitive with good layer penetration. Sample cleanliness less crucial	Scans of complex geometries is more difficult. Signal interpretation is complicated	Yes	No	Med	Currently deployed system for monitoring corrosion and coatings in this application
Infrared thermography	Thickness, general integrity of coating, defects and flaws	Size of detectable flaw limited	Easy collection and characterisation of data. Can be used on large areas. Does not always require heating input	Limited sensitivity and resolution. Prone to environmental noise	Yes	Yes	High–Med	Promising lab testing for this application has been carried out
Pulsed thermography	Thickness, general integrity of coating, defects and flaws	Size of detectable flaw limited	Can detect a large number of flaws. Rapid imaging	Limited by non-uniform heating effects. Requires clean surface and increases in expense as resolution increases	Yes	No	High–Med	Promising lab testing for this application has been carried out
Eddy current pulsed thermography	Thickness, general integrity of coating, defects and flaws	Size of detectable flaw limited	Quick and easier heating method than other thermography	Limits of sensitivity	Yes	No	High–Med	Promising lab testing for this application has been carried out
Corrosion indicating paint	pH, production of oxide or other species	Why coating has failed	No equipment and can be implemented easily into any paint system	Only a reactive technology. May be unsightly for a customer. May require a change in paint application or production methods	No	No	High–Med	Promising lab testing for this application has been carried out. Small scale deployment has been achieved
Stressed optical fibres	Decrease in thickness deformation or movement of coating	Small flaws, onset of corrosion	Instantaneous data response. Estimation of lifetime possible	Manufacturing difficulties. Effect on paint integrity. Reactive technology. May not detect some failure modes	Yes	Yes	Med	Currently a deployed system for monitoring corrosion in other applications. Promising lab testing for this application has been carried out
Bragg gratings	Deformation from blisters or delamination. Cracking or defect formation	Changes in colour	Well developed technology. High sensitivity and can survive coating processes	Difficulty in embedding sensors. Fibres are delicate and could break under mechanical action. Temperature dependent	Yes	Yes	Low	Currently a deployed system for monitoring corrosion in other applications. Promising lab testing for this application has been carried out
Corrosion product detection	Corrosion product	Flaws, delamination or coating integrity	Allows monitoring of rate of corrosion. Can determine coating failure	Affected by a number of factors. Only a reactive technology and relatively newly developed. Requires sensitive calibration	Yes	Yes	Med–Low	Currently an early-stage technique for monitoring corrosion in this application
Interferometer	Thickness measurements, delamination, displacements	Porosity of coating, optical changes	Very accurate distance measurements	Requires calibration and very sensitive so prone to noise. Difficulty in embedding in coating	Yes	Yes	Med–Low	Currently a deployed system for monitoring corrosion in other applications. Promising lab testing for this application has been carried out
Corrosion coupons	Rate of corrosion	Coating integrity or failure method	Easy to use and install. Can indicate regions of concern and be used to estimate lifetimes	Requires time and analysis. Requires isolation from external factors. Prone to reading errors and human error	No	No	High	Currently deployed system for monitoring corrosion and coatings in this application
Galvanic	Rate of corrosion	Coating integrity or failure method	Easy to use and install. Can indicate regions of concern and be used to estimate lifetimes	Requires time and analysis. Requires isolation from external factors. Prone to reading errors and human error	No	No	High	Currently deployed system for monitoring corrosion and coatings in this application
Zero resistance ammetry	Rate of corrosion	Coating integrity or failure method	Easier data analysis as it does not require weight measurements. Low cost and simple	Limited application to coated steels currently. May only be useful for atmospheric corrosivity	Yes	Yes	Med	Currently a deployed system for monitoring corrosion in other applications. Promising lab testing for this application has been carried out
Electrical resistance	Rate of material loss, rate of corrosion	Coating integrity or failure method. Well developed technology	Low maintenance, reliable and easy to operate and interpret	Requires environmental considerations. Temperature and pressure dependant. Assumes uniform corrosion on sample	Yes	Yes	Med	Currently a deployed system for monitoring corrosion in other applications. Promising lab testing for this application has been carried out
Induction resistance probes	Rate of material loss, rate of corrosion	Coating integrity or failure method	Improved sensitivity over ER	Requires magnetic materials. Shorter probe life than ER	Yes	Yes	Med	Currently a deployed system for monitoring corrosion in other applications. Promising lab testing for this application has been carried out
Capacitive	Thickness of coating, permittivity	Location of defects	Wide range of coating information can be gained. Better than eddy current or magnetic methods for coatings	Requires connection to base substrate	Yes	Yes	High–Low	Currently a lab technique for monitoring corrosion and coatings in this application. Efforts are being made to produce deployed systems
Electrical field signature method (FSM)	Thickness changes, substrate damage, cracks	Non-conductive layers	Can detect local and general corrosion. Good for measuring large areas	Requires complex analysis/referencing. Sensitive and delicate. May not be applicable to coatings	Yes	Yes	Med	Currently a deployed system for monitoring corrosion in other applications. Developmental lab testing for this application has been carried out
Potentiodynamic polarisation	Rate of corrosion	Coating integrity or failure method	Directly related to corrosion kinetics	Requires electrolyte submersion. Gives instantaneous value of corrosion not cumulative damage. Does not measure coating	Yes	Yes	Low	Currently a lab technique for monitoring corrosion and coatings in this application. Efforts are being made to produce deployed systems
Harmonic analysis	Current, rate of corrosion	Coating integrity or failure method	Directly related to corrosion kinetics. Less assumptions than LPR	Requires electrolyte submersion.Gives instantaneous value of corrosion not cumulative damage. Does not measure coating	Yes	Yes	Med	Developmental lab testing for this application has been carried out
Electrochemical impedance spectroscopy	Thickness, permittivity	Location or severity of defects	Very commonly used for coating analysis. Can be miniaturised. Gives consistent dat.	Complex data analysis which is prone to noise and poor data fitting	Yes	Yes	Med	Currently a lab technique for monitoring corrosion and coatings in this application. Efforts are being made to produce deployed systems
Electrochemical noise	Rate of corrosion, location of defects	Types of defects or severity	Does not interfere with corrosion process. Consistent with EIS measurements. Has been successfully applied to coatings	Does not indicate mode of corrosion. Sensitive to interference and noise	Yes	Yes	Med	Currently a lab technique for monitoring corrosion in other application. Efforts are being made to produce deployed systems
Scanning electrochemical microscopy	Spatially resolved reactivity and rate of degradation	Coating integrity or failure method	Gives an indication as to the chemical species involved. Locational accuracy and high resolution	Requires complex movement control. Would be difficult to attempt on a large scale. Is affected by surface cleanliness	Yes	No	Low	Currently a lab technique for monitoring corrosion in this application
Strain gauges	Coating delamination, blistering or other movement	Degradation not resulting in strain	Sensitivity, ease of data handling. Can be printed onto substrates	Requires embedding in coating. Difficulty of receiving information. Requires calibration and is prone to damage	Yes	Yes	Med	Currently a lab technique for monitoring corrosion in this application. Efforts are being made to produce deployed systems
MEMS-based sensors	Coating movement or strain. Changes in polarisation or chemical concentration	Some features may be too complex to accurately measure	Versatile well developed technology. Small and cheap	Requires embedding in coating. Difficulty of receiving information. Little currently focused on coating monitoring	Yes	Yes	Med–Low	Currently a deployed system for monitoring in other applications. Developmental lab testing for this application has been carried out
Thin layer activation	Degradation of coating as a barrier. Loss of material	Size of detectable flaw limited	Material unaffected by irradiation. Well correlated with mass lost. Easy to scale up	Requires irradiation. Can be affected by many external factors. Manual sensing required. May not be directly linked to coating	Yes	Yes	Low	Developmental lab testing for this application has been carried out
RFID	Theoretically any information could be transferred	Difficulty in transferring large quantities of data	May not require power. Can relay information passively. Allow easy data collection and analysis	Difficulties of embedding. Complex circuit required. May not be able to function passively	Yes/No	No	Med–Low	Currently a deployed system for other sectors. Promising lab testing for this application has been carried out
NFC	Theoretically any information could be transferred	Simpler reader system than RFID	Provides easy passive data extraction at low cost	Difficulty of metal environment and combining with sensors	No	No	Med–Low	Currently a deployed system for other sectors. Promising lab testing for this application has been carried out
Bio-detectors	Conditions for MIC	Coating integrity or failure method	Allows determination of maintenance scheduling. Indicates areas for concern	Does not actually measure coating performance directly. Extrapolative method	Yes	Yes	Med–Low	Currently a deployed system for other sectors. Promising lab testing for this application has been carried out
pH sensors	pH change	Coating integrity or failure method	Detect early onset of corrosion. The technology is well developed for other industries	May require electrolyte submersion. Difficulties embedding in coating. Not applied frequently to coatings	Yes	Yes	Med–Low	Currently a deployed system for other sectors. Promising lab testing for this application has been carried out
Chemical sensors	Products of corrosion or by-products of coating degradation	Very localised so may not reflect the overall performance	May not require power. May give insight into failure method and or integrity	Difficulty in embedding sensors. May require immersion. Delicate circuitry or chemistry involved. Manufacturing difficulties	Yes/No	Yes	Med–Low	Currently a deployed system for other sectors. Promising lab testing for this application has been carried out

## Data Availability

Not applicable.
